# Benchmarking computational methods for identifying and quantifying polyadenylation sites from 3′ tag-based single-cell RNA-seq data

**DOI:** 10.1093/nar/gkag490

**Published:** 2026-05-12

**Authors:** Xingyu Bi, Zhen Chen, Mengmeng Ye, Tao Zhang, Danni He, Xiaohui Wu

**Affiliations:** Department of Hematology, Children’s Hospital of Soochow University, Suzhou 215000, China; Cancer Institute, Suzhou Medical College of Soochow University, Suzhou 215000, China; Cancer Institute, Suzhou Medical College of Soochow University, Suzhou 215000, China; Cancer Institute, Suzhou Medical College of Soochow University, Suzhou 215000, China; Cancer Institute, Suzhou Medical College of Soochow University, Suzhou 215000, China; Cancer Institute, Suzhou Medical College of Soochow University, Suzhou 215000, China; Department of Hematology, Children’s Hospital of Soochow University, Suzhou 215000, China; Cancer Institute, Suzhou Medical College of Soochow University, Suzhou 215000, China; Suzhou Key Laboratory of Pathogen Bioscience and Anti-infective Medicine, MOE Key Laboratory of Geriatric Diseases and Immunology, Suzhou 215000, China; Jiangsu Key Laboratory of Infection and Immunity, Soochow University, Suzhou 215000, China; Pediatric Hematology and Oncology Key Laboratory of Higher Education Institutions in Jiangsu Province, Suzhou 215000, China; Biomedical Basic Research Center in Jiangsu Province, Suzhou215000, China

## Abstract

Alternative polyadenylation (APA) is a widespread post-transcriptional regulatory mechanism in eukaryotes, which contributes greatly in shaping transcriptome complexity and proteome diversity. The advancement of 3′ tag-based single-cell RNA sequencing (scRNA-seq) technology has facilitated the emergence of various computational methods for identifying and quantifying polyadenylation sites (pAs) at the single-cell level. However, the lack of benchmarking complicates the choice of a suitable method. Here, we systematically benchmarked 10 methods using 9 simulated datasets and 25 real-world scRNA-seq datasets covering four sequencing protocols and three species of animals and plants. First, we proposed strategies based on prior pA annotations and base compositions around pAs to evaluate the sensitivity and accuracy of different methods on identifying pAs. Particularly, we evaluated the consistency of pA identification results across different methods, as well as the quality of unique pAs identified by each method. Furthermore, we assessed the performance of pA quantification of different methods using strategies based on correlation coefficients, cell type clustering, and differential APA detection. Finally, we evaluated computational resource consumption for each method. Practical guidelines were provided for choosing suitable methods, particularly with respect to factors such as sensitivity and accuracy in pA identification and quantification, the availability of prior pA annotations, scRNA-seq protocols, and species.

## Graphical Abstract

**Graphical Abstract:**
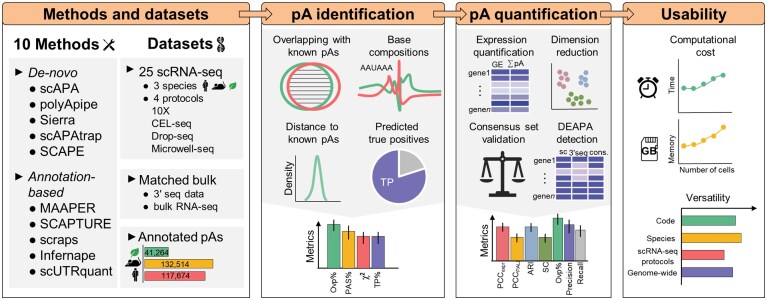

## Introduction

Alternative polyadenylation (APA) is a prevalent post-transcriptional regulatory mechanism in eukaryotes, which involves selecting different poly(A) sites (hereinafter called pAs) in pre-messenger RNA (mRNA) to generate mRNA isoforms with distinct coding regions or 3′ untranslated regions (3′ UTRs). APA contributes to enhancing the transcriptome complexity and the intricate dynamics of gene regulation for eukaryotes [1–3]. APA has been observed in ~70% of mammalian genes [4, 5], playing an important regulatory role in various biological processes such as cell proliferation, differentiation [6–8], and tumorigenesis [9–11].

In recent years, advancements in single-cell RNA sequencing (scRNA-seq) technology have opened up new avenues for exploring APA events at the single-cell level. Several 3′ tag-based scRNA-seq protocols that enrich mRNA 3′ ends using poly(A) primers, such as 10X Chromium [12], CEL-seq [13], Drop-seq [14], and Microwell-seq [15], can generate sequence reads enriched at the 3′ ends of transcripts, which can be applied to APA analysis. Since 2019, various computational methods specifically designed to identify pAs from scRNA-seq data have emerged [16–18]. Here, we divided these methods into two categories according to their underlying strategies. The first category involves peak-calling-based methods used for *de novo* pA identification, including scAPA [19], Sierra [20], scAPAtrap [21], SCAPE [22], and polyApipe (used in the PASTA module [23] that interfaces directly with Seurat). The fundamental principle of these methods is that reads originating from different RNA molecules of the same transcript isoform pile up to a peak at the 3′ end. The second category comprises methods that rely on existing pA annotations and can be further divided into two subclasses according to their capacity to uncover novel pAs: (i) “read-support filtering of annotated pAs,” illustrated by MAAPER [24] and scUTRquant [25], and (ii) “peak calling followed by annotation filtering,” exemplified by SCAPTURE [26], Infernape [27], and scraps [28]. The principle of these methods is to utilize existing pA annotation information as a reference to predict pAs or validate the reliability of identified pAs. Although these numerous methods have been proposed to detect and quantify pAs from 3′ tag-based scRNA-seq data, principles and applicable data of these methods vary, and the studies corresponding to different methods often use limited datasets or evaluation metrics for performance assessment, posing challenges for benchmarking these methods. Currently, there are several benchmarking studies on methods for pA identification and quantification [29–34]. For example, Shah *et al*. [30] conducted a benchmark evaluation on APA analysis methods for bulk RNA-seq protocols, demonstrating that the accuracy of these methods was inferior to those using 3′-end sequencing (3′ seq) and Iso-seq data as input. Bryce-Smith *et al*. [31] also conducted a benchmark study on methods for bulk RNA-seq data. They benchmarked eight methods using a comprehensive set of RNA-seq experiments comprising real, synthetic, and matched 3′ seq data. Subsequently, Tian *et al*. [32] benchmarked five pA prediction methods and seven differential APA detection methods across five RNA-seq datasets, revealing significant differences in accuracy, sensitivity, and consistency among methods. However, these studies only focused on the comparison of methods for bulk RNA-seq. Recently, Li *et al*. [33] compared data characteristics that could affect APA detection performance across seven 3′ tag-based scRNA-seq protocols, and evaluated the ability of six computational methods to identify and quantify pAs using simulated and real scRNA-seq data. However, simulated data may not be able to fully capture the complex and noisy peak features present in real data, such as overlapping peaks and peaks heavily influenced by sequencing artifacts. Furthermore, Li *et al*. only utilized data from a single species of mouse and compared only six methods, which limited the universality and applicability of the evaluation results. Lately, Zhao *et al*. [34] evaluated the performance of six methods on one 10X Chromium dataset and three spatial transcriptomics datasets from mouse and validated them using paired ONT (Oxford Nanopore Technologies) data. However, this study also suffers from the deficiency of insufficient evaluation data and methods. In addition, they only evaluated pAs on genes commonly identified by different methods, while ignoring unique pAs identified by a single method, which may result in deviations in the evaluation results. Specifically, current benchmark studies were limited to species with abundant and easily accessible pA resources, such as human or mouse, which restricts the applicability of the assessment results to a wider range of species, such as plants. Moreover, most studies did not assess the applicability and performance of the methods under different scRNA-seq protocols. Therefore, it is necessary to use broader datasets from different species and scRNA-seq techniques to conduct more comprehensive and systematic benchmarking of the numerous methods currently available.

In this study, we benchmarked 10 pA identification methods using 9 simulated datasets and 25 real datasets covering four scRNA-seq protocols and three animal and plant species. We proposed several strategies based on prior pA annotations and base compositions around pAs to evaluate the sensitivity and accuracy of different methods on identifying pAs. Particularly, we evaluated the consistency of pA identification results across different methods, as well as the quality of unique pAs identified by each method. Furthermore, we assessed the performance of pA quantification of different methods. Specifically, we compared the correlation coefficients between the pA expression levels quantified by individual methods from scRNA-seq data and the pA expression levels of matched 3′-seq data at both the pA and gene levels. Moreover, we evaluated the effects of pA expression profiles and APA usage profiles obtained from different methods on downstream cell-type clustering. In addition, we evaluated the performance of differentially expressed APA (DEAPA) detection based on pA quantification results from different methods. Finally, we evaluated computational resource consumption for each method. Our results would provide users with general guidelines for choosing optimal methods for pA identification and quantification, and also promote the development and optimization of APA research methods in the field of single-cell transcriptomics.

## Materials and methods

A brief overview of the materials and analytical procedures is provided below. Detailed descriptions are available in the Supplementary Text.

### Data collection and preprocessing

To ensure a comprehensive and representative evaluation, we compiled public benchmark datasets as listed in Supplementary Table 2, including 25 public scRNA-seq datasets across four protocols (15 10X Chromium, six CEL-seq, two Drop-seq, two Microwell-seq), spanning multiple species and technical variations. Additionally, we collected matched 3′-seq and bulk RNA-seq data (Supplementary Table 2) to evaluate the accuracy of APA quantification and DEAPA gene identification.

### Existing computational methods for pA identification and quantification

This study benchmarked 10 methods (Supplementary Table 1), classified into two categories: (i) *de novo* pA identification methods, including scAPA [19], polyApipe [23], Sierra [20], scAPAtrap [21], and SCAPE [22]; and (ii) methods based on prior pA annotations, including MAAPER [24], SCAPTURE [26], Infernape [27], scUTRquant [25], and scraps [28]. All methods were executed following official tutorials with default parameters. Due to differences in supported species and sequencing protocols, some methods failed on specific datasets (Supplementary Table 3). For *Arabidopsis* analyses requiring reference pAs, we used custom-curated annotations. SCAPTURE’s DeepPASS model does not support *Arabidopsis*, Infernape could not generate pA expression counts under default settings, and scUTRquant requires a prebuilt *Arabidopsis* transcriptome target; thus, these three methods were not applied to real *Arabidopsis* data.

### Annotation of pAs

First, low-expression pAs were filtered, retaining only those expressed in ≥0.5% of cells. Internal priming-derived false pAs were removed using species-specific filtering strategies from each method. For tools outputting exact pA coordinates, genomic regions were annotated directly; for those outputting peak ranges, intervals were collapsed to representative sites (e.g. mean position for SCAPE; 3′-most base for Infernape, scAPA, and other related callers) before assessment. To ensure consistency, all pAs were uniformly annotated using the annotatePAC function in movAPA [35]. Annotated 3′ UTRs were extended by 2000 bp (human, mouse) and 1000 bp (*Arabidopsis*) to capture nearby downstream pAs. Since scAPA uses hg19 GENCODE [36], its called sites were converted to hg38 via UCSC LiftOver prior to annotation.

### Collection of the reference pA datasets

**Figure 1.**
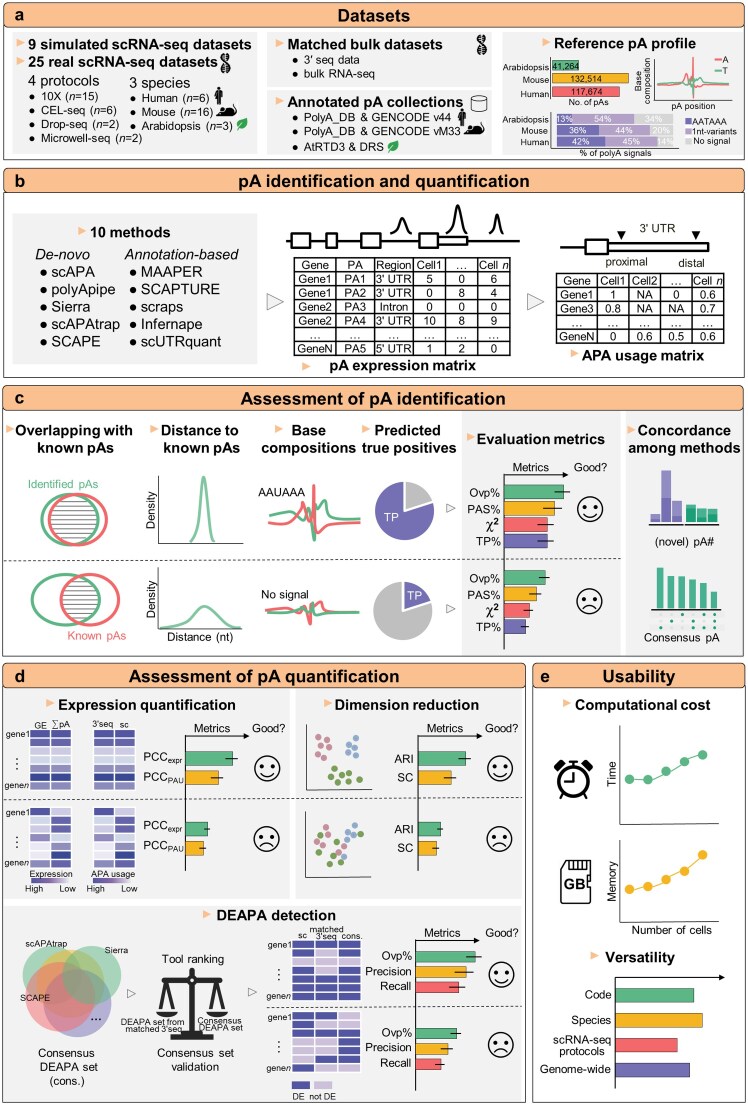
Schematic overview of the benchmarking workflow. (**a**) Summary of the simulated and real scRNA-seq datasets, annotated pAs, and matched bulk datasets used in this study. (**b**) The 10 pA identification and quantification methods can be categorized into two groups based on whether they rely on prior pA annotations: *de novo* and annotation-based. Based on pAs identified by each method, the pA expression matrix and APA usage matrix can be obtained. (**c**) Performance evaluation of different methods for pA identification. Several strategies based on prior pA annotations and base compositions around pAs were used to evaluate the sensitivity and accuracy of different methods. The consistency of pA identification results across different methods, as well as the quality of unique pAs identified by each method, were also evaluated. (**d**) Assessment of the performance of pA quantification of different methods using strategies based on correlation coefficients, cell-type clustering, and DEAPA detection. A strategy was proposed to construct consensus DEAPA gene set as a reference, and the reliability of this strategy was validated using DEAPA results from matched 3′-seq data. (**e**) Evaluation of the computational resource consumption for each method, and summarization of the usability and generality of different methods. pA, polyadenylation site; PAS, poly(A) signal; Ovp, overlapping; TP, true positive; PAU, pA usage; PCC, Pearson correlation coefficient; ARI, adjusted rand index; SC, silhouette coefficient.

We collected known pA annotations as ground truth for evaluation. Human and mouse pA annotations were obtained from GENCODE (v44 and vM33) [36] and polyA_DB 3 [37], with the latter lifted over from hg19/mm9 to hg38/mm10 using UCSC LiftOver. *Arabidopsis* pA annotations were taken from previous studies [38, 39]. Following a published method [40], we integrated cross-source pA records for each species and unified all references to hg38, mm10, and TAIR10. Using movAPA [35], we annotated pAs and extended 3′ UTRs to generate final reference sets, yielding 117 674 human, 132 514 mouse, and 41 267 *Arabidopsis* pAs (Fig. 1a).

### Benchmarking on simulated data

We generated 3′ tag-based simulated scRNA-seq data with known APA events and realistic peak features following Li *et al*. [33]. Using real data from human PBMCs, mouse sperm, and *Arabidopsis* root cells, we produced three biological replicates per species.

We assessed pA quantification accuracy by computing the mean absolute percentage error (MAPE) between predicted and ground-truth expression matrices at both barcode and group levels. At the barcode level, MAPE was defined as follows:


(1) \begin{eqnarray*}
\mathrm{ MAP}{{\mathrm{ E}}_{\mathrm{barcode}\ }} = \ \frac{1}{{n\ \times m}}\ \mathop \sum \limits_{j = 1}^n \mathop \sum \limits_{i = 1}^m \frac{{\left| {{{c}_{i,j}} - {{{\hat{c}}}_{i,j}}} \right|}}{{{{c}_{i,j}}}}.
\end{eqnarray*}

Here *n* is the number of barcodes, and *m* is the number of matched pAs (only those with non-zero ground truth expression are included). The absolute percentage error for pA *i* in barcode *j* is calculated as $\frac{{| {{{c}_{i,j}} - {{{\hat{c}}}_{i,j}}} |}}{{{{c}_{i,j}}}}$, where ${{c}_{i,j}}$ is the ground truth expression value and ${{\hat{c}}_{i,j}}$ is the predicted expression value. At the group level, MAPE was calculated based on the total expression of each pA across all cells.


(2) \begin{eqnarray*}
\mathrm{ MAP}{{\mathrm{ E}}_{\mathrm{group}}} = \ \frac{1}{m}\ \mathop \sum \limits_{i = 1}^m \frac{{\left| {c_i^{\mathrm{total}} - \hat{c}_i^{\mathrm{total}}} \right|}}{{c_i^{\mathrm{total}}}}.
\end{eqnarray*}

Here *m* is the number of matched pAs (only those with non-zero ground truth total expression are included). The absolute percentage error for pA *i* across all cells is calculated as $\frac{{| {c_i^{\mathrm{total}} - \hat{c}_i^{\mathrm{total}}} |}}{{c_i^{\mathrm{total}}}}$, where $c_i^{\mathrm{total}}$ is the ground truth total expression value and $\hat{c}_i^{\mathrm{total}}$ is the predicted total expression value.

### Evaluation of pA identification

We developed a chi-square metric to evaluate nucleotide composition similarity around predicted and reference pAs. We calculated A/T/C/G frequencies from 100 nt upstream to 100 nt downstream of pAs, used the R chisq.test to obtain per-position statistics, and took the average as the similarity score, where smaller values indicated higher similarity. We then compared base frequencies with the reference and derived a representative statistic.

To quantify how sequencing depth influences pA detection, we used samtools to down-sample the 146-million-read mouse-sperm BAM to 10%, 30%, 50%, 70%, and 90% read fractions, reran each method on the reduced files, annotated the resulting 3′ UTR pAs, and compared the depth-dependent yield across methods.

### Evaluation of unique pAs

The agreement score [41] was used to assess the consistency of pA identification across different methods within a given dataset (equation 3).


(3) \begin{eqnarray*}
{\mathrm{Agreement}} = \frac{1}{{\left| S \right|}}\mathop \sum \limits_{pAi\in S} \frac{{{{n}_{pAi}} - 1}}{{N - 1}}.
\end{eqnarray*}

Here, *N* denotes the number of pA identification methods, *S* denotes the total number of pAs, consisting of all pAs identified by all methods, and ${{n}_{pAi}}$ is the number of methods identifying pA *i*. The metric ranges from 0 to 1, where a value of 0 means that pA sets from all methods are completely distinct, and a value of 1 means that pA sets from all methods are the same.

Unique pAs of each method were defined as sites detected by no other approach and absent from the reference pA set. To validate these sites, we employed DeepPASTA [42], a robust deep-learning model that has maintained top-tier pA prediction performance in recent benchmarks [26]. Using 200 nt flanking sequences and RNA secondary structure, we applied DeepPASTA to score unique pAs in human and mouse data, yielding prediction probabilities from 0 to 1. Sites with probability >0.5 were classified as positive, and we calculated the fraction of positive unique pAs for each method.

### Evaluation of pA quantification

#### Evaluation of pA quantification at the gene level

To compare gene-level quantification across methods, we summed pA expression counts per gene. For 10X Chromium, Microwell-seq, CEL-seq, and Drop-seq data, gene-level references were generated using Cell Ranger, STAR, and STARsolo, respectively. We then computed Pearson correlations between method-derived and reference gene expression for common genes.

We also evaluated quantification via cell-type clustering across 17 annotated datasets (Supplementary Table 2). Clustering performance was compared using gene expression, pA expression, and APA usage, where APA usage was defined as the relative usage of the distal site (RUD) per gene per cell. We used the external metric ARI (Adjusted Rand Index) and the internal metric SC (Silhouette Coefficient) to assess clustering accuracy, respectively.

#### Evaluation of pA quantification at the pA level

We evaluated pA quantification consistency by calculating Pearson correlations of total expression for consensus pAs (within 24 nt) between method pairs. High-confidence pAs (identified by all methods) were analyzed in 12 datasets with >200 such sites.

Using matched 3′-seq and bulk RNA-seq data as ground truth, we assessed PAU [Poly(A) site usage] quantification by comparing proximal/distal PAU differences. Known pAs were used where available; otherwise, pAs were quantified by polyAseqTrap [43] or QAPA [44].

To avoid bias, we selected APA genes detected by ≥6 methods and supported by bulk/3′ seq data. Proximal and distal pAs were defined by movAPA, and ΔPAU values were computed per method. Pearson correlations were calculated using the top 60% genes with the smallest ΔPAU deviations from ground truth.

### Evaluation of DEAPA gene identification

#### Construction of the consensus DEAPA gene set

As true DEAPA genes are unknown in real datasets, we developed a multi-level weighted strategy to build a consensus DEAPA gene set for evaluation. Detailed procedures are as follows:

(i) DEAPA gene identification

To evaluate DEAPA gene identification, we applied two differential analyses: DEXseq and the Wilcoxon rank-sum test. For DEXseq, cells were randomly divided into six pseudo-bulk replicates per group to detect differentially used APA sites (adjusted *P*-value < .05, |FC| > 1.5), with corresponding genes defined as candidate DEAPA genes. For the Wilcoxon test, we used three APA usage metrics to identify genes with differential APA usage.

(ii) Standardization and ranking

For each gene, we ranked them in ascending order of adjusted *P*-value and descending order of |log_2_FC| based on the pAs with the most significant differences, and calculated the PR (Percentile Rank).

(iii) Determination of regulatory direction

We determined the regulatory direction of DEAPA genes (3′ UTR lengthening or shortening) to avoid conflicting trends across methods. For each gene, pAs were ranked by distance to the stop codon (rank 1 = proximal, others = distal). Only the pA with the largest |log2FC| was used to assign direction: proximal pA with log_2_FC < 0 indicated lengthening; distal pA with log_2_FC < 0 indicated shortening. This classified DEAPA genes into lengthened and shortened subsets.

(iv) Calculation of the WF score

For each DEAPA gene, we counted supporting methods and computed a weighted frequency (WF) score using PR: each contributing method added a value of (1 − PR). Genes with WF ≥1.6 and supported by ≥ four methods were defined as final consensus DEAPA genes.


(4) \begin{eqnarray*}
W{{F}_{\mathrm{gene}}} = \mathop \sum \limits_{t = 1}^T \left( {1 - P{{R}_{\mathrm{gene},t}}} \right) \times I\left( {{\mathrm{DEAP}}{{{\mathrm{A}}}_{\mathrm{gene},t}}} \right).
\end{eqnarray*}

$P{{R}_{\mathrm{gene},t}}\ $ represents the significance percentile rank of a gene in the differential analysis results obtained from the pA expression counts quantified by the *t*th method. *I*$({\mathrm{DEAP}}{{{\mathrm{A}}}_{\mathrm{gene},t}})\ $is an indicator function that takes the value of 1 if the *t*th method identifies a gene as a DEAPA gene; otherwise, it takes the value of 0. The WF score ranges from [0, *T*] (where *T* is the total number of methods) and represents the composite confidence level for a gene consistently identified as a DEAPA gene by multiple methods. A higher score indicates a greater likelihood that the gene is a genuine DEAPA gene.

#### Metrics for evaluating DEAPA gene identification

We used the consensus DEAPA gene set as the ground truth and evaluated the accuracy of detected DEAPA genes by calculating precision and recall. To assess consistency in DEAPA gene identification between methods, we calculated the Jaccard index among DEAPA genes and the Spearman correlation coefficient of their significance rankings.

## Results

### Overview of the benchmarking pipeline

To evaluate computational methods for APA detection in scRNA-seq data, we established a comprehensive framework integrating simulated and real datasets (Fig. 1). We benchmarked ten methods (Supplementary Table 1) using 9 simulated data and 25 real scRNA-seq datasets spanning four scRNA-seq protocols and three species (Supplementary Table 2), enabling holistic performance assessment across ideal and practical scenarios.

For real dataset benchmarking, we used curated reference pA annotations and matched bulk 3′ seq/RNA-seq data as evaluation standards (Fig. 1a). The 10 methods were divided into *de novo* (scAPA, polyApipe, etc.) and annotation-based (MAAPER, SCAPTURE, etc.) groups (Fig. 1b). Post pA identification, uniform annotation was performed to generate pA expression and APA usage matrices (Fig. 1b).

To compare pA identification performance (Fig. 1c), we used annotated pAs as references to quantify distances to detected pAs, analyzed poly(A) signal distributions for biological plausibility, and applied a chi-square metric to assess single-nucleotide profile similarity. Moreover, a deep learning model estimated true positive rates for novel pAs, and we evaluated inter-method consistency and uniquely detected pA quality.

Next, we assessed pA expression quantification performance (Fig. 1d) by calculating correlation coefficients between scRNA-seq-derived expression and two references (original gene expression, 3′ seq-derived pA expression), and examined the impact of pA/APA profiles on downstream cell type clustering.

Furthermore, we evaluated the performance of DEAPA gene detection based on pA quantification results from different methods. We constructed a consensus DEAPA gene set from all methods, validated against 3′ seq results. We also evaluated computational resource consumption across datasets and summarized tool usability to provide practical guidance (Fig. 1e).

### Benchmarking pA identification and quantification methods using simulated data

We first generated nine simulated datasets, mirroring the key features of real single-cell sequencing data from human, mouse, and *Arabidopsis*, to establish a controlled baseline for evaluating pA identification accuracy. We assessed performance across a window size range of 0–100 nt (step 10 nt), defining a predicted pA as a true positive if it fell within this range of a known site.

**Figure 2.**
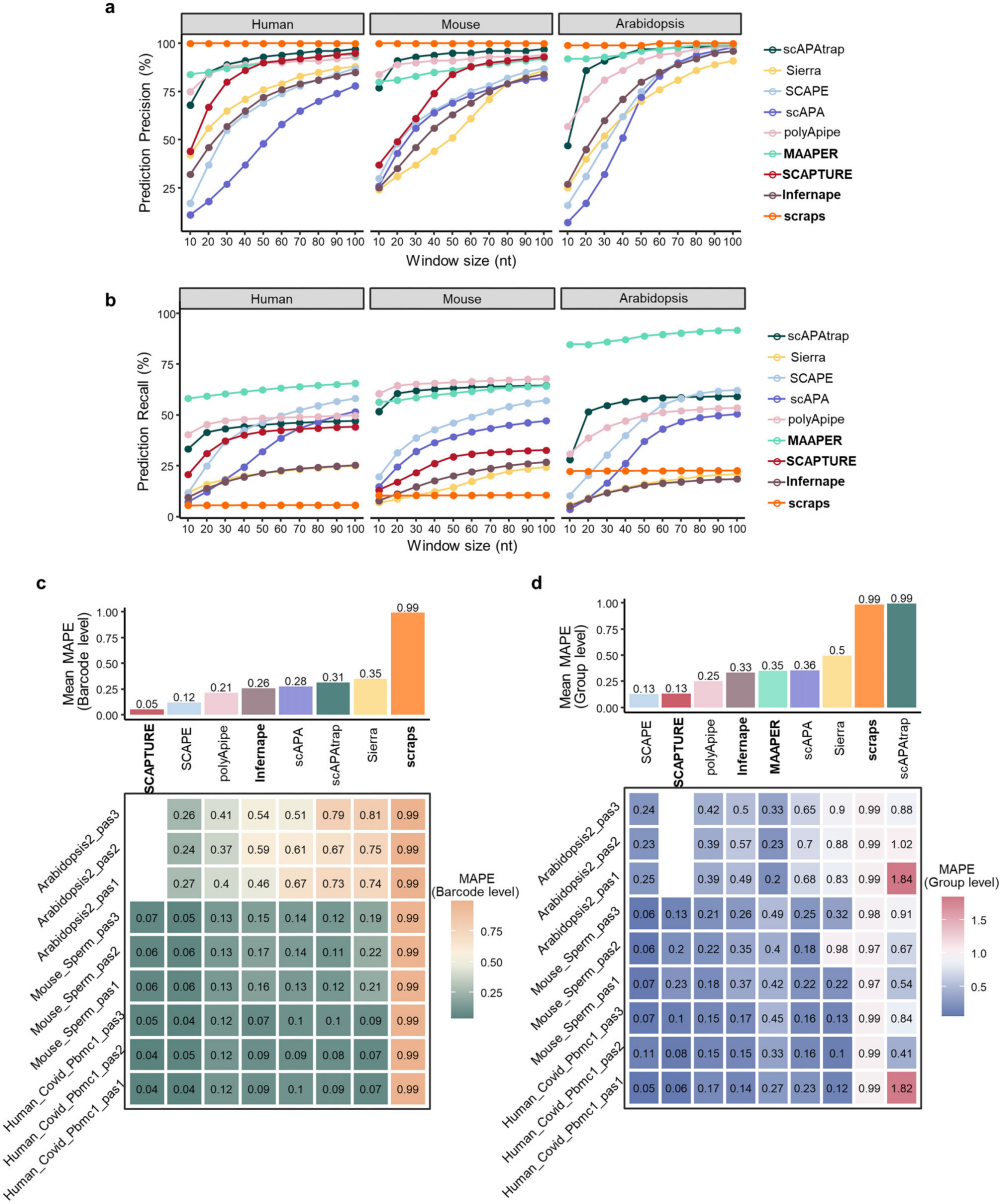
Evaluation of methods for pA identification and quantification using simulated scRNA-seq datasets. (**a**) Average precision of pA prediction across simulated datasets derived from different species. The *x*-axis indicates the window size (nt); a predicted pA was labeled as a true positive if it fell within the specified window distance from any annotated ground-truth pAs. The *y*-axis represents precision, defined as the fraction of predicted pAs that matched true pAs. (**b**) Average recall of pA prediction across simulated datasets from different species, with the same window-size definition as in panel (a). The *y*-axis represents recall, defined as the fraction of annotated ground-truth pAs that were successfully recovered by each method. (**c**) MAPE of pA expression quantification at the single-cell barcode level across datasets. Only pAs that could be matched to ground-truth annotations were included in this analysis. Lower MAPE values reflect higher quantification accuracy. MAAPER was excluded from the barcode-level MAPE calculation because it only outputs aggregate pA expression levels at the cell-population level rather than single-cell resolution. (**d**) MAPE of pA expression quantification at the cell-group level across datasets, with the same site-matching criterion as in panel (c).

Most methods exhibited a consistent trend: precision and recall rose initially with increasing window size, then plateaued (Fig. 2a and b). At the narrowest 10 nt window, scraps showed near-perfect precision but extremely low recall, indicating high localization accuracy but severe under-detection. In contrast, MAAPER, polyApipe, and scAPAtrap delivered balanced performance, exceeding 85% precision at a 50 nt window while maintaining high recall. Across all three species, these three methods demonstrated the most robust and comprehensive pA identification.

We further evaluated quantification accuracy by calculating the MAPE between method-predicted and known ground-truth expression, at both barcode and cell-group levels (using a 24 nt matching threshold [45, 46]). Results revealed significant performance variation by species and scale (Fig. 2c and d): SCAPTURE and SCAPE outperformed all others with consistently low MAPE across species, demonstrating strong robustness. scraps showed high errors at both levels, while scAPAtrap performed moderately at the barcode level but saw a sharp MAPE increase at the group level, likely due to amplified quantification biases during cell aggregation.

Additionally, most methods achieved lower errors in human/mouse than *Arabidopsis* data, and errors were generally lower at the barcode level, with accumulation at the group level. To investigate this, we recalculated MAPE using only pAs identified by at least six methods. Strikingly, focusing on these multi-method consensus sites reduced group-level MAPE to near barcode levels (Supplementary Fig. 1). This indicates elevated group-level error stems from low-abundance, low-stability true pAs identified by few methods. Their single-cell quantification errors compound during aggregation, inflating MAPE. Conversely, consensus pAs are highly expressed, broadly distributed, and stable, ensuring low, consistent errors at both scales.

While simulated data provides a definitive ground truth, it lacks the complexity of real sequencing data (e.g. overlapping peaks, artifacts, coverage variation). Thus, we further conducted a comprehensive benchmark using real datasets spanning multiple species and sequencing protocols.

### Benchmarking computational methods for predicting pAs

**Figure 3.**
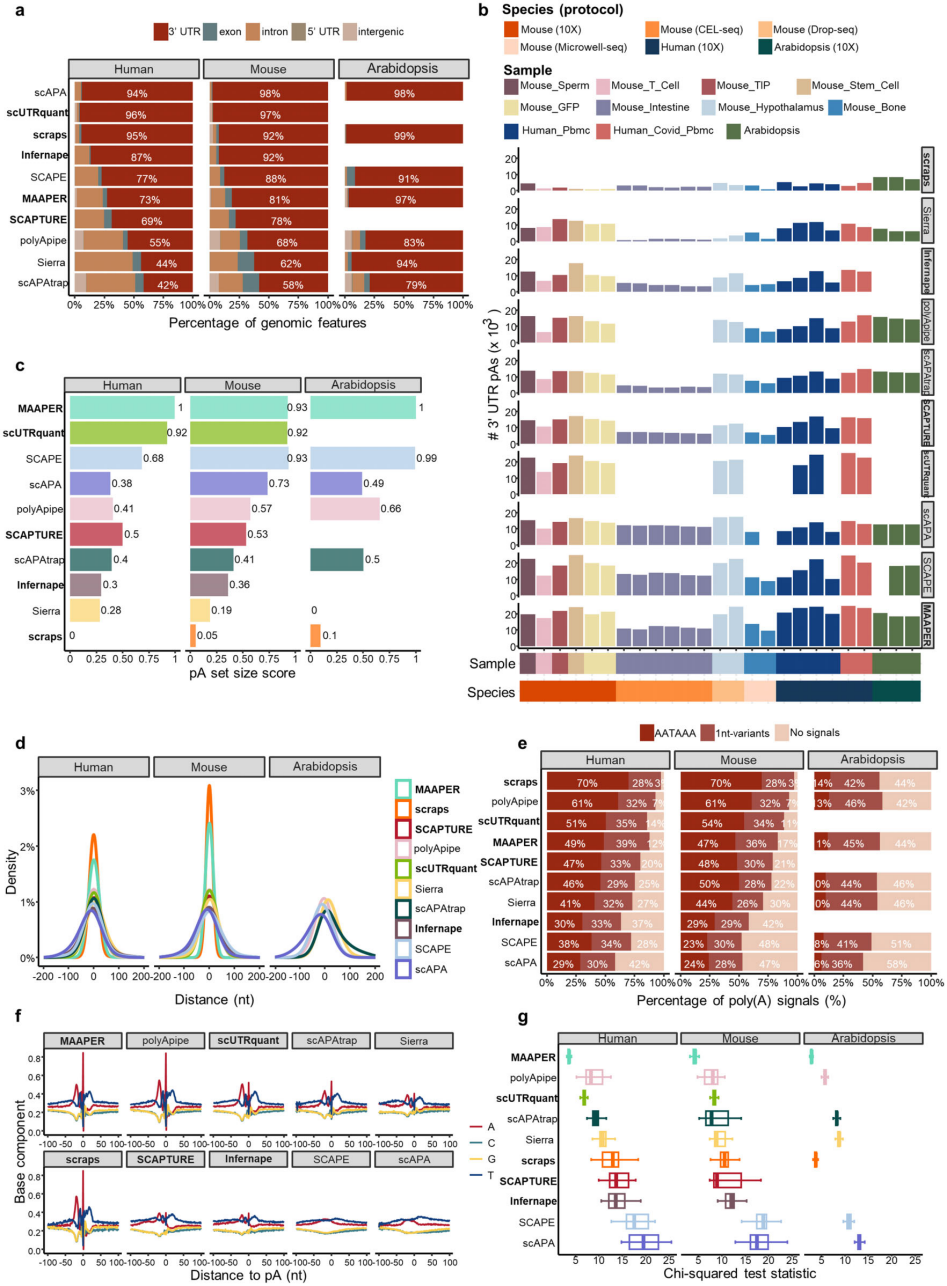
Evaluation of methods for pA identification. (**a**) Distribution of genomic region of pAs identified by different methods across datasets from different species. The method name in bold indicates a method based on prior pA annotations, while others are *de novo* methods. (**b**) Number of 3′ UTR pAs identified by each method across 25 scRNA-seq datasets from three species and four sequencing protocols. (**c**) The average pA set-size score of each method in datasets from different species, with a higher score indicating a greater number of identified pAs. (**d**) Distance distribution between 3′ UTR pAs identified by each method and reference pAs. For the *Arabidopsis* data, MAAPER and scraps were run with the identical pA annotation used to construct the reference set; consequently, every predicted site coincides exactly with a reference entry, yielding a 100% offset frequency at zero. Hence, their density curves collapse to an infinitesimally narrow spike that is invisible in the plot. (**e**) The average proportion of 3′ UTR pAs containing poly(A) signal motifs identified by each method for datasets from different species. (**f**) Nucleotide compositions of the sequences surrounding 3′ UTR pAs identified by each method in the mouse sperm cell data. *Y*-axis denotes the fractional nucleotide content at each position. *X*-axis denotes the position, and 0 is the position of the pA. (**g**) Boxplots showing scores of chi-squared metric of 3′ UTR pAs identified by each method. The smaller the chi-square value, the more consistent the single nucleotide profile between the identified pAs and the reference ones (Supplementary Fig. 2).

We utilized the 10 methods to identify pAs from 25 scRNA-seq datasets spanning three species. First, we examined the distribution of pAs identified by each method across genomic regions (Fig. 3a). Several methods, such as scAPAtrap, Sierra, polyApipe, and SCAPTURE, can identify pAs across multiple regions, while others like scAPA, scUTRquant, scraps, Infernape, and SCAPE, are restricted to 3′ UTR. Specially, scAPAtrap and polyApipe can also detect intergenic pAs. Since 3′ UTR pAs are universally recognizable, subsequent analyses focused on this subset.

Quantification of 3′ UTR pA numbers revealed substantial method-dependent variability (Fig. 3b). On most datasets, scraps identified the fewest pAs, while MAAPER and SCAPE consistently detected the highest numbers. Performance also varied by scRNA-seq protocol. For instance, in the CEL-seq Mouse_Intestine dataset, polyApipe and scUTRquant failed, and Sierra identified the fewest number of pAs, even fewer than scraps. Furthermore, we proposed the pA set size score (see “Materials and methods” section) as a metric to quantify the sensitivity of each method in detecting pAs (Fig. 3c). MAAPER consistently identified the most 3′ UTR pAs across most datasets of different species, followed by scUTRquant and SCAPE. Meanwhile, scraps and Sierra again ranked lowest.

Next, we used collected pA annotations (Fig. 1a) as the ground truth to evaluate pA location accuracy. The distance to the nearest reference pA (Fig. 3d) showed that annotation-based methods generally yielded more precise locations than *de novo* methods. Among annotation-based methods, scraps and MAAPER were closest to references, with nearly all pAs within 50 nt. MAAPER’s strict adherence to annotations maximized precision but limited novel pA discovery. Among *de novo* methods, pAs identified by polyApipe were the closest to the reference pAs. Plant datasets generally showed larger distances to references than animal datasets, likely due to less comprehensive plant annotations or greater polyadenylation micro-heterogeneity [47].

Moreover, we assessed the presence of poly(A) signals in the upstream 50 nt (Fig. 3e). For human and mouse datasets, over 80% of pAs identified by scraps, polyApipe, scUTRquant, and MAAPER were signal-supported, while for *Arabidopsis* datasets, this dropped to ∼55%. In comparison, scAPA, SCAPE, and Infernape identified a relatively lower proportion of pAs containing poly(A) signals.

Finally, we analyzed single-nucleotide profiles (100 nt flanking regions) and their similarity to references. Taking the mouse sperm cell dataset as an example (Fig. 3f), except for SCAPE and scAPA, the single-nucleotide profiles of most methods are generally close to the reference profile (Supplementary Fig. 2), with MAAPER, polyApipe, and scraps appearing to be the closest. We further utilized a chi-square score (see “Materials and methods” section) to quantify the similarity of the single nucleotide profile of identified pAs with that of reference pAs (Fig. 3g). MAAPER, polyApipe, and scUTRquant achieved the lowest chi-square score (most similar to reference), while SCAPE and scAPA scored the highest.

These results indicate that annotation-dependent methods (e.g. scraps, MAAPER, and scUTRquant) exhibit superior pA localization accuracy than *de novo* methods. However, polyApipe, as a *de novo* method, can also obtain relatively accurate pAs, comparable to methods based on pA annotations.

### Benchmarking computational methods for pA prediction across scRNA-seq protocols

**Figure 4.**
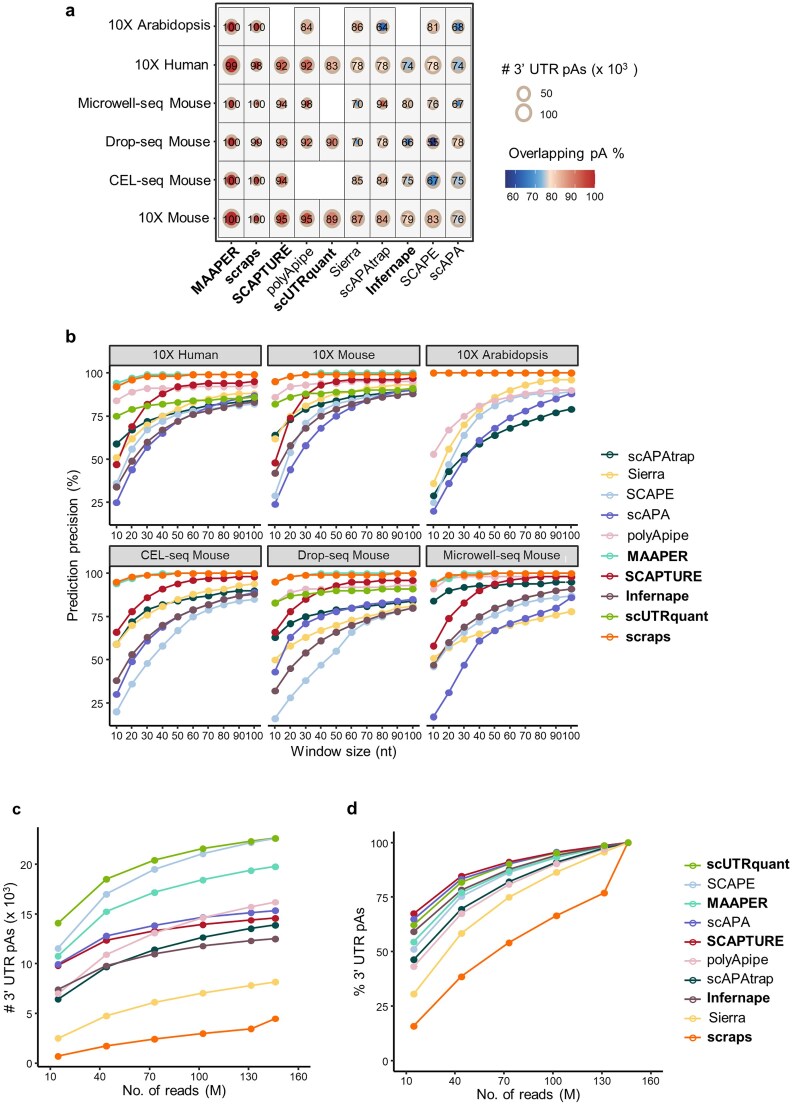
Evaluation of the performance of pA identification of different methods on data from different scRNA-seq protocols. (**a**) Comparison of the overlap rates between pAs identified by different methods and reference pAs across datasets from different sequencing protocols. pAs falling within the 50 nt region of any reference pAs were defined as overlapping. Color indicates the proportion of overlapping pAs, and circle size represents the total number of identified pAs. (**b**) Average precision of pAs identified by each method across datasets from different sequencing protocols. pAs falling within a given window size (nt) of any reference pAs were defined as true. Number (**c**) and percentage (**d**) of 3′ UTR pAs of each method versus sequencing depth, obtained by down sampling a 146-million-read mouse sperm dataset.

Next, we evaluated the performance of pA identification of different methods on data from different scRNA-seq protocols. First, we calculated the proportion of method-identified pAs overlapping with reference pAs across protocols (Fig. 4a). Most annotation-based methods—except Infernape—ranked among the top for overlap rate. Notably, the *de novo* method polyApipe achieved comparable overlap rate, though it was inapplicable to CEL-seq data. While scraps also had a high overlap rate, it identified the fewest pAs overall. As an annotation-based method, MAAPER can be deployed only when a pre-compiled pA annotation is provided; yet it is precisely this enforced reliance on prior knowledge that enables it to deliver the highest overlap rate among all methods.

We further assessed precision across protocols using window sizes (0–100 nt) to measure pA alignment with references. As expected, precision increased with window size before plateauing (Fig. 4b), and scRNA-seq protocols significantly impacted method performance. For example, on Microwell-seq dataset, methods relying on soft-clipped poly(A) stretches (polyApipe, scAPAtrap) outperformed others; this is because ∼35% of Microwell-seq reads span the mRNA 3′ cleavage site [15, 48], aiding accurate pA identification—unlike other protocols with few untemplated adenosines. Conversely, SCAPE performed poorly on CEL-seq and Drop-seq data, likely due to mismatched empirical insert-size parameters (optimized for 10X Chromium/Microwell-seq) causing pA location offsets. Notably, MAAPER, scraps, SCAPTURE, and polyApipe performed consistently well on applicable datasets: MAAPER and scraps reached near-100% precision at 30 nt, while SCAPTURE and polyApipe exceeded 84% precision at 50 nt. However, regardless of the window size, SCAPE and scAPA exhibited relatively poor prediction precision. Supplementary analyses of single-nucleotide profiles and poly(A) signal motif proportions further supported these findings (Supplementary Figs 3 and 4).

Given substantial sequencing depth variation across protocols (Supplementary Table 2), we next tested depth dependence using a deeply sequenced mouse sperm dataset (146 million reads), down-sampling to measure pA yield decline. When coverage dropped to 15 million reads, scUTRquant, SCAPE, and MAAPER retained high pA identification sensitivity (Fig. 4c)—attributable to their pA identification principles (predefined pAs for scUTRquant; read-to-pA distance estimation for SCAPE/MAAPER). While all methods identified fewer pAs with decreasing depth, SCAPTURE, scAPA, and scUTRquant showed relatively smaller declines, indicating greater robustness to coverage variations (Fig. 4d). In contrast, Sierra and scraps were highly depth-sensitive: Sierra’s coverage-fitting approach and scraps’ reliance on poly(A)-tailed reads led to sharp pA yield drops at low coverage. These results indicate that pA identification methods perform differently across scRNA-seq protocols and sequencing depths, highlighting the need to select tools tailored to the specific sequencing protocol used.

### Evaluating the consistency of pA identification results across different methods

**Figure 5.**
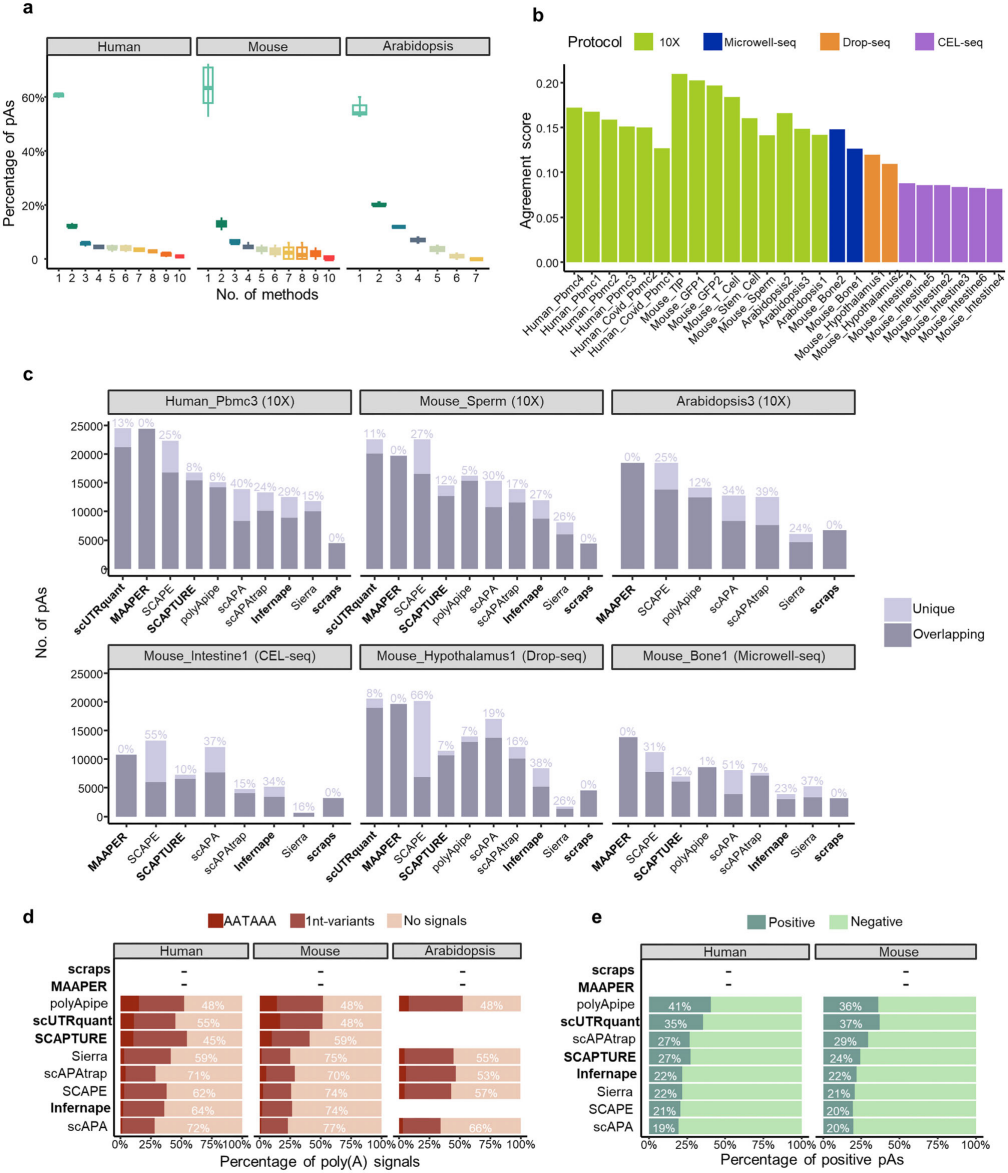
Evaluation of unique pAs identified by different methods. (**a**) Proportion of pAs that are commonly identified by different numbers of methods for the data from different species. pAs identified by different methods were pooled if they were located within 24 nt of each other. (**b**) Agreement score (ranging from 0 to 1) that measures the concordance of pAs identified by different methods for the same dataset. (**c**) The number and proportion of unique pAs identified by different methods in six representative datasets from different species and sequencing protocols. (**d**) The average percentage of unique pAs containing poly(A) signal motifs identified by each method in datasets from different species. The results of MAAPER and scraps are not displayed because they identified too few unique pAs (<50). (**e**) The average percentage of unique pAs that were predicted as positive by DeepPASTA.

Having evaluated the accuracy of pAs identified by each method, we next assessed the consistency of pA calls across methods. Most pAs were identified by only a single method, while very few were identified by all methods (Fig. 5a). We calculated an agreement score (0–1) to quantify inter-method consistency (Fig. 5b). Overall scores were low across all datasets (0.08–0.21), revealing substantial divergence in pA calls. Notably, CEL-seq datasets showed the lowest agreement, indicating fewer shared pAs across methods under this protocol.

We speculated that low consistency may reflect method-specific false positives. We therefore extracted unique pAs—sites detected by only one method and absent from reference annotations—and quantified their number and proportion (Fig. 5c). Compared with annotation-based methods (excluding Infernape), *de novo* methods, especially SCAPE and scAPA, identified far more unique pAs. By contrast, scraps, which relies on both soft-clipped poly(A) reads and annotations, yielded very few unique pAs (only 4 in human and 12 in mouse). Since MAAPER strictly anchors predictions to annotated sites, it produced only a very small number of unique pAs.

To assess the authenticity of unique pAs, we examined the presence of poly(A) signals within 50 nt upstream of unique pAs (Fig. 5d) and employed DeepPASTA [42] to predict true-positive status from flanking sequences (Fig. 5e). Across mammals, poly(A) signals are highly conserved [49], so the human-trained model was applied to mouse. We observed a clear positive correlation between poly(A) signal enrichment and DeepPASTA-predicted validity. The proportion of unique pAs containing poly(A) signals identified by annotation-based methods scUTRquant and SCAPTURE is relatively higher compared to most *de novo* methods except for polyApipe. Over 52% of unique pAs identified by polyApipe contained poly(A) signals, and 41% and 36% of unique pAs were predicted as positive for human and mouse datasets, respectively. In contrast, over 62% of unique pAs identified by SCAPE and scAPA did not contain any poly(A) signal for human and mouse datasets, and only ~20% of unique pAs were predicted by DeepPASTA as positive.

Together, these results indicated that annotation-based methods (excluding Infernape) and the *de novo* method polyApipe generate fewer but more reliable unique pAs, whereas other *de novo* tools predict numerous unique sites with lower credibility.

### Benchmarking computational methods for quantifying pAs

**Figure 6.**
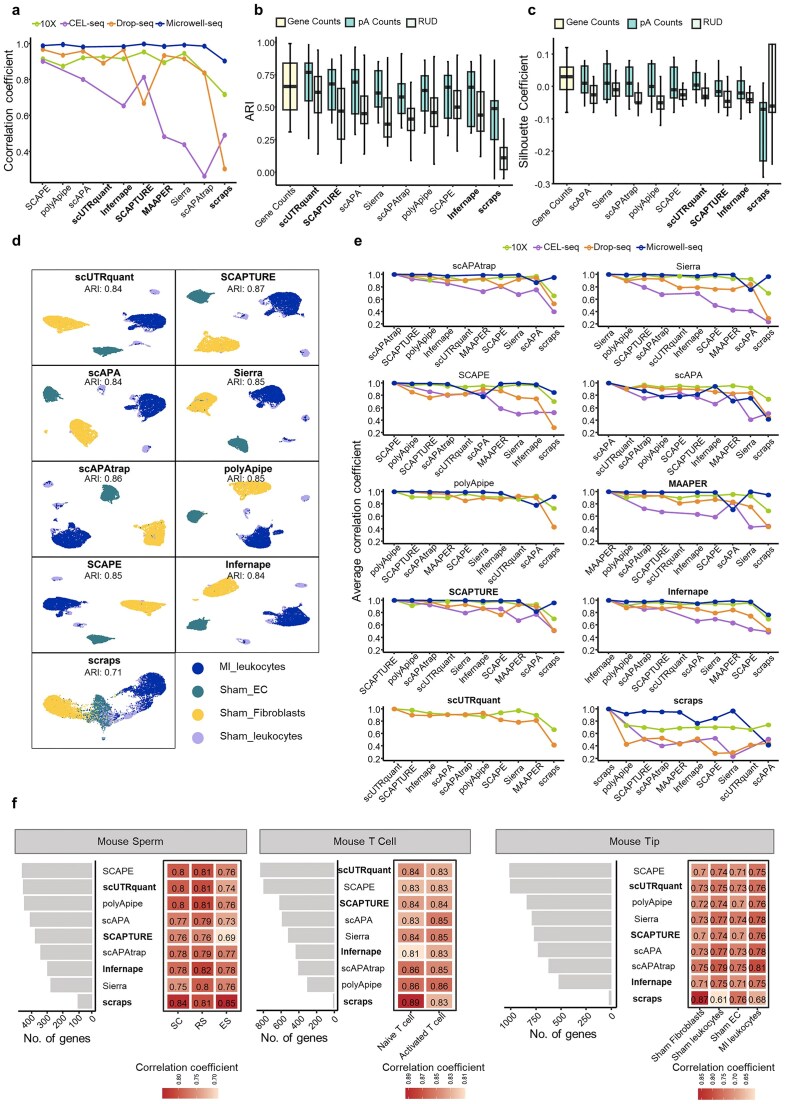
Evaluation of methods for pA quantification. (**a**) The Pearson correlation coefficient between gene expression counts and the sum of read counts of pAs quantified by different methods for common genes across datasets from different scRNA-seq protocols. (**b**) ARI scores of cell type clustering based on gene expression, pA expression, and APA usage measured by relative usage of the distal pA (RUD) in 17 datasets from 10X Chromium and Microwell-seq. (**c**) Same as b, except that SC scores were calculated. (**d**) UMAP plots based on the pA expression profiles obtained by different methods for the mouse TIP data. (**e**) The average Pearson correlation coefficients of consensus pAs between a specific APA method and all other methods across datasets from different scRNA-seq protocols. (**f**) The Pearson correlation coefficients between the △PAU estimated by different methods in a cell type for common APA genes identified from scRNA-seq and matched bulk data. For mouse sperm and mouse T cell samples, the matched 3′ seq data were used as the ground truth; for mouse TIP sample, the matched bulk RNA-seq data were used as the ground truth. The left figure shows the number of common APA genes used for calculating the correlation, and the right figure shows the correlation. Since MAAPER only generated the total expression level of pAs in a cell population, it was not included in cell clustering, calculation of △PAU, and DEAPA analysis.

After evaluating the accuracy and sensitivity of pA identification, we next assessed the performance of each method in pA quantification. Since total expression level of pAs within a gene approximates gene-level expression, we calculated correlations between method-derived pA-based gene expression and original gene expression across all 25 datasets. All methods achieved high correlations on Microwell-seq data, followed by 10X Genomics data (Fig. 6a). On Drop-seq data, most methods performed well except SCAPTURE and scraps. For CEL-seq data, SCAPE, SCAPTURE, and scAPA achieved relatively high correlations, whereas other methods performed poorly, likely due to low mapping rates that distorted pA and gene-level quantification.

Next, we used 17 datasets with cell type annotation information to evaluate cell type clustering results based on pA profiles quantified by each method. Clustering was performed separately using pA expression levels and RUD (Relative Usage of the Distal pA) values. Using annotated cell types as the ground truth, we evaluated clustering performance with the ARI (Adjusted Rand Index) and SC (Silhouette Coefficient) (Fig. 6b and c; Supplementary Fig. 5). With the exception of scraps, which detected far fewer pAs, clustering based on pA expression profiles yielded ARI values comparable to or even higher than those from gene expression profiles (Fig. 6b). By contrast, clustering using RUD values generally produced lower ARI and SC scores across all methods, likely because RUD profiles carry less transcript-level information than full pA expression profiles. UMAP visualization confirmed that pA expression profiles from methods with high ARI scores clearly separated distinct cell types (Fig. 6d and Supplementary Fig. 6), whereas scraps lacked sufficient pA coverage to reliably distinguish cell populations.

We further compared pairwise correlations of consensus pA expression across methods for each scRNA-seq protocol (Fig. 6e). Overall, polyApipe, SCAPTURE, scAPAtrap, and scUTRquant showed high correlation with most methods except scraps. Notably, SCAPTURE and scAPAtrap maintained strong correlations across all four protocols; polyApipe performed similarly across three protocols but not CEL-seq; and scUTRquant showed high consistency on 10X and Drop-seq data. Correlations among methods were generally high on 10X and Microwell-seq data (excluding scraps), but much lower on CEL-seq data, especially for scraps, Sierra, and MAAPER.

We also examined quantification consistency for consensus pAs detected by all ten methods. In 10X Chromium datasets with sufficient high-confidence pAs, pairwise expression correlations among most methods exceeded 0.9, indicating strong consistency (Supplementary Fig. 7). We further evaluated APA usage quantification by comparing △PAU (differences in proximal/distal PAU) estimated from scRNA-seq with bulk 3′ seq or RNA-seq reference data (Fig. 6f). To avoid bias, we selected shared APA genes identified by at least six methods and supported by bulk/3′ seq data for this comparison. Most methods achieved correlations near 0.8 across datasets, indicating consistent APA quantification for shared high-confidence APA genes. However, the number of overlapping APA genes varied widely: scUTRquant and SCAPE identified the most, while scraps detected dramatically fewer (Fig. 6f).

In summary, our benchmark demonstrates that SCAPE, scUTRquant, SCAPTURE, and polyApipe perform favorably in pA quantification at both gene and pA levels, with SCAPE and polyApipe showing particularly strong cross-platform robustness.

### Benchmarking computational methods for detecting DEAPA genes

Changes in relative expression between APA sites often reflect biologically relevant differences. We therefore evaluated each method’s ability to identify DEAPA genes using eight datasets with cell type annotations. Since scRNA-seq lacks definitive DEAPA ground truth and matched bulk 3′ seq data is limited, we developed a strategy to construct a consensus DEAPA gene set (top DEAPA genes identified by ≥4 methods) as a reference for validation (see “Materials and methods” section).

We first validated this consensus strategy using mouse sperm and T cell scRNA-seq data with matched bulk 3′ seq data. Method rankings for DEAPA identification were consistent whether using the scRNA-seq-derived consensus set or 3′ seq-derived DEAPA genes as the reference (Supplementary Fig. 8a). Notably, a large portion of scRNA-seq DEAPA genes identified by each method were not detected in matched 3′ seq data (Supplementary Fig. 8b), indicating that 3′ seq alone cannot fully evaluate scRNA-seq DEAPA results. Thus, our consensus-based reference strategy is both effective and necessary.

**Figure 7.**
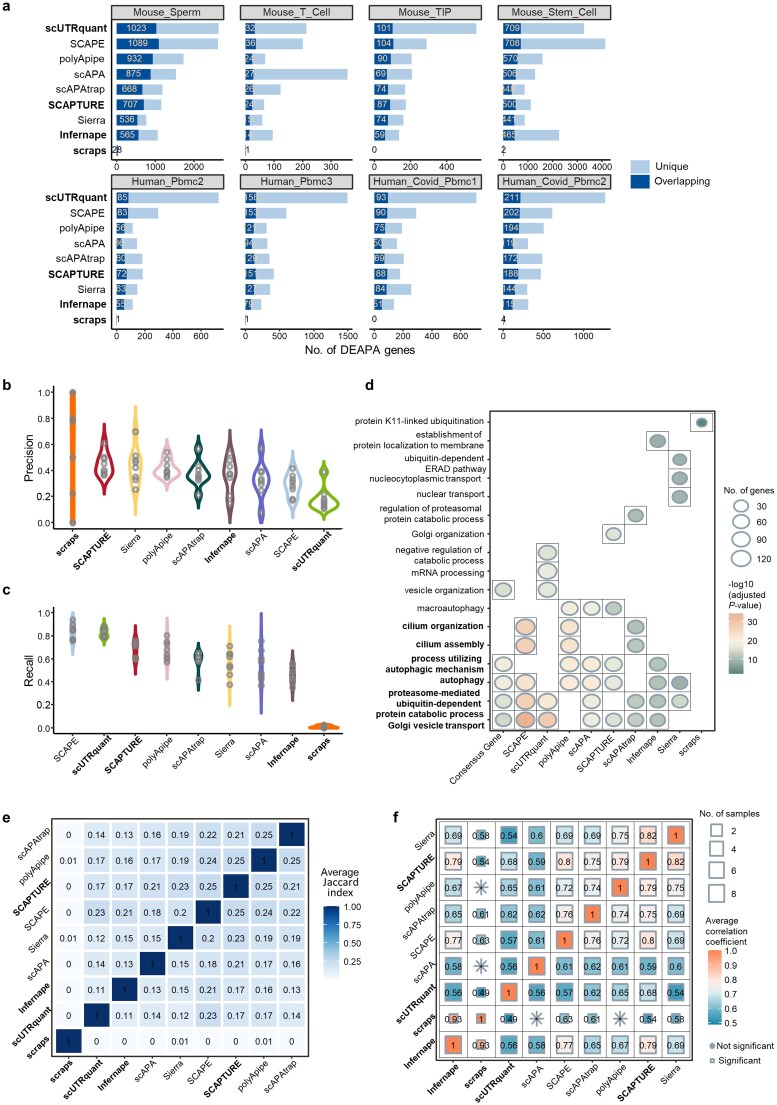
Evaluation of methods for DEAPA analysis. (**a**) The number of DEAPA genes identified based on the pA profile obtained by each method across different datasets, and the number of genes validated by the consensus DEAPA gene set. (**b**) Comparison of the precision of DEAPA results of different methods. (**c**) Comparison of the recall of DEAPA results of different methods. (**d**) Gene Ontology (GO) analysis results based on DEAPA genes identified by each method as well as the consensus DEAPA genes for the mouse sperm cell data. A maximum of top five significantly enriched biological processes are shown. The biological process highlighted in bold has been widely proven to play an important role in spermatogenesis. (**e**) The average Jaccard index for DEAPA genes identified by different methods. Values closer to 1 indicate higher consistency. (**f**) The Spearman correlation coefficient of significance rankings between DEAPA genes identified by different methods, with rankings determined by the adjusted *P*-value of the pA with the largest |log_2_FC| value on DEAPA genes. The size of the squares indicates the number of datasets used to calculate the correlation coefficient (i.e. data with at least ten overlapping DEAPA genes between methods and adjusted *P*-value < .05). If two methods identify ≥ 10 overlapping DEAPA genes in a single dataset but the adjusted *P*-value of the correlation coefficient is ≥ .05, the result is marked with ※ to indicate non-significant correlation. Color indicates the strength of the correlation.

We next counted DEAPA genes identified by each method that overlapped with the consensus set (Fig. 7a). Similar to 3′ seq results, only a moderate proportion of method-identified DEAPA genes were validated by the consensus set, suggesting each method uniquely detects a substantial number of DEAPA genes. For precision (Fig. 7b) and recall (Fig. 7c): scraps achieved the highest precision but the lowest recall (due to identifying the fewest genes); SCAPE, scUTRquant, polyApipe, and SCAPTURE showed strong recall, but only polyApipe and SCAPTURE balanced precision and recall effectively. Supplementary analyses using the Wilcoxon rank-sum test on three APA usage indices—RUD, RUP (Relative Usage of the Proximal pA), and PSI (*ψ*)—confirmed that top-performing methods maintained stable advantages across different differential analysis strategies, validating their quantification reliability (Supplementary Fig. 9).

To assess biological relevance, we performed GO enrichment analysis on method-identified and consensus DEAPA genes. Using mouse sperm cell differentiation as an example, most method-identified DEAPA genes were significantly enriched in key spermatogenesis pathways (Golgi vesicle transport, proteasome-mediated ubiquitin-dependent protein catabolism, autophagy, cilium assembly/organization)—processes critical for acrosome biogenesis, protein homeostasis, and flagella assembly [51–54] (Fig. 7d). Method-specific differences emerged in enrichment strength: SCAPE, scUTRquant, and scAPA showed higher significance in Golgi vesicle transport and proteasome pathways, while SCAPE, polyApipe, and SCAPTURE enriched more strongly in autophagy and cilium assembly. The consensus set, while significantly enriched in most key pathways, missed some functionally relevant genes in processes like cilium assembly, highlighting the value of integrating multi-method results for comprehensive APA functional analysis.

Finally, we evaluated inter-method consistency in DEAPA detection using the Jaccard index and correlation of DEAPA significance rankings (Fig. 7e and f). SCAPE, SCAPTURE, and polyApipe showed the highest consistency, confirming their reliability for DEAPA gene identification.

### Run time and memory usage

We evaluated computational resource usage of each method during pA identification and quantification using five representative mouse scRNA-seq datasets with ∼55 000 genes and varying cell numbers. All analyses were performed on an Ubuntu 22.04.5 LTS system equipped with an Intel(R) Xeon(R) Gold 6248R CPU (3.00 GHz, 24 cores) and 256 GB RAM. For multithreaded steps, all methods were consistently run using 12 threads.

**Figure 8.**
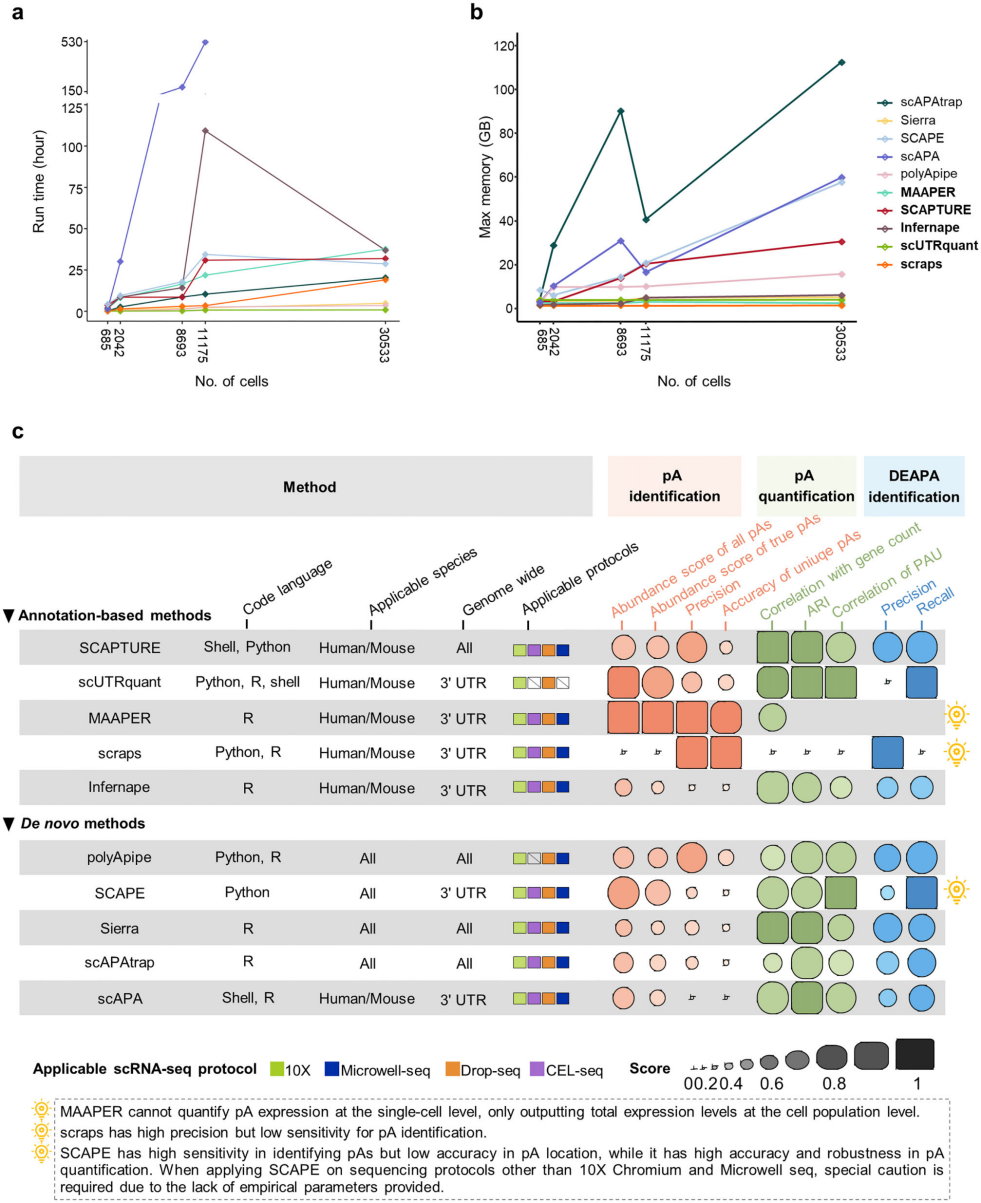
Comparison of time and memory consumption of different methods, as well as summary of evaluation results. (**a**) Run time (in hours) of each method under different number of cells. Since scAPA takes longer to identify and quantify pAs when processing datasets with larger number of cells, we did not record the total time required for the successful run in datasets with >30 000 cells, but only the maximum memory consumed before stopping the run. (**b**) Max memory consumption (in GB) of each method under different number of cells. Since scAPAtrap consumes a large amount of memory to search tails for large genome, when the number of cells exceeds 10 000, the large BAM file needs to be split into four BAM files by chromosomes to avoid process termination due to insufficient memory. Since scAPA ran for >21 days when processing datasets with >30 000 cells, we failed to record the total time it took to run successfully, but only the maximum memory consumed before it stopped running. (**c**) Summary of characteristics and performance of different methods. Performance scores for each method under each evaluation metric were based on the results on the real 10X Chromium datasets for human and mouse.

In terms of runtime, scUTRquant, polyApipe, and Sierra were significantly faster than other tools, whereas scAPA exhibited the longest execution time (Fig. 8a). The high computational cost of scAPA stems from its strategy of splitting BAM files by individual cell barcodes during pA quantification, making its runtime highly sensitive to increasing cell numbers.

For peak memory consumption, MAAPER, Sierra, Infernape, scUTRquant, and scraps maintained relatively low memory usage (Fig. 8b). Memory requirements for MAAPER, Sierra, and Infernape were largely unaffected by increasing cell numbers, while scraps and scUTRquant remained stable, likely due to their batch-processing designs. In contrast, memory usage by SCAPTURE, SCAPE, scAPA, and scAPAtrap rose substantially with larger cell populations. Notably, scAPAtrap in genome-wide tail-search mode (tails.search = genome) is particularly memory-intensive, and we recommend splitting large BAM files into smaller chunks when analyzing >10 000 cells to reduce memory load.

### Summary of the methods’ performance

Our benchmarking results demonstrate that no single method outperforms others across all datasets and metrics; optimal selection depends on experimental objectives and data characteristics (Fig. 8c).

*For pA identification*: Annotation-based methods (except Infernape) generally achieved higher location accuracy. While scraps identified pAs with high precision, it sacrificed sensitivity and detected far fewer pAs than other tools (Fig. 3c). Among *de novo* methods, polyApipe balanced sensitivity and accuracy most effectively. SCAPE detected more pAs but had a high false-positive rate, requiring cautious use (Figs 3c and 4a). Inter-method consistency in pA identification was low, with most methods detecting numerous unique pAs (Figs 5a and b); of these, polyApipe identified a moderate number of unique pAs with higher poly(A) signal proportions and true-positive rates, demonstrating superior accuracy and reliability (Fig. 5d and e).

*For pA quantification*: SCAPE, scUTRquant, SCAPTURE, and polyApipe exhibited superior accuracy, with SCAPE and polyApipe showing strong cross-platform robustness (Fig. 6). Notably, while MAAPER achieved high sensitivity and accuracy in pA identification, it only quantified pAs at the cell population level, not single-cell resolution.

*For DEAPA gene identification*: SCAPE and scUTRquant detected more DEAPA genes but had higher false positives, whereas SCAPTURE and polyApipe achieved an optimal balance of precision and recall (Fig. 7b and c). Importantly, pA identification accuracy was independent of quantification performance: e.g. SCAPE had lower pA identification accuracy but maintained good quantification accuracy and robustness.

Sequencing protocols also influenced method performance (Figs 4 and 6); for instance, most methods performed poorly on CEL-seq data for both pA identification and quantification.

In summary, for high-confidence pA identification, annotation-based methods (excluding scraps and Infernape) are preferred. For exploratory analysis, polyApipe is recommended, as it is suitable for data from any species. Considering overall performance across pA identification, quantification, and DEAPA analysis, we recommend the annotation-based method SCAPTURE and the *de novo* method polyApipe as the optimal choices.

## Discussion

In this study, we investigated the performance of different methods on data from four 3′ tag-based scRNA-seq protocols including 10X Chromium, CEL-seq, Drop-seq, and Microwell-seq. Overall, most methods generally performed well on 10X Chromium data but poorly on CEL-seq data (Figs 4b, 5b, and 6a, e). This may be because the 10X platform is widely applied and has mature alignment algorithms. Consequently, most methods prioritize adaptation to 10X Chromium data during algorithm design. Secondly, the low sequencing depth of the CEL-seq dataset used in this study resulted in insufficient coverage of reads on the genome. This significantly impacted methods that predict pAs by fitting coverage changes, such as Sierra. Notably, the proportion of reads spanning mRNA 3′ end cleavage sites obtained from Microwell-seq is higher than other sequencing technologies. This facilitates methods relying on soft-clipped poly(A) stretch algorithms (e.g. polyApipe and scAPAtrap) to achieve higher prediction accuracy compared to other sequencing datasets. However, such methods are particularly sensitive to changes in read length and are not suitable for short-read data. SCAPE is a distance-based estimator that calls pAs from insert-size and tail-length distributions. Default empirical parameters are supplied for 10X Chromium and Microwell-seq, and in the absence of protocol-specific calibrations for other platforms, we retained these defaults. Nevertheless, library-preparation chemistry, fragmentation bias and poly(A)-tail distributions differ markedly across protocols, so the shipped parameters may not be universally optimal. Protocol-specific re-estimation or optimization of the insert-size and tail-length priors is therefore advisable before deploying SCAPE on new data types. In summary, the performance of most methods is affected by scRNA-seq platform and data characteristics. Researchers should select appropriate methods based on the sequencing read length, coverage, and data quality of their experimental data, and pay attention to the compatibility limitations of the methods for the target sequencing protocols.

In terms of pA identification, we evaluated sensitivity and accuracy of different methods. Regarding sensitivity, we observed that scraps identified significantly fewer pAs compared to other methods (Fig. 3b). This is because, except for the Microwell-seq dataset, all datasets are single-end aligned, and scraps relies solely on read 2-only alignments that end with a soft-clipped poly(A) stretch to infer pAs for single-end alignment data. However, <15% of read 2 alignments contain soft-clipped poly(A) sequences, resulting in lower resolution and sensitivity, and the loss of a significant amount of pAs information. Moreover, we found that some methods showed significant differences in sensitivity across different species. For example, both scAPA and SCAPE exhibited markedly reduced sensitivity of pA identification in human datasets compared to mouse (Fig. 3c). Furthermore, scUTRquant and SCAPTURE are currently not applicable to *Arabidopsis* data due to the lack of pA annotations in required format and trained models for species other than mouse or human, respectively. In terms of accuracy of pA identification, previous benchmarking studies [22, 34] used ONT data as a ground truth for evaluation, leveraging the advantage of full-length reads in transcript identification. However, ONT data typically have lower sequencing depth and quality compared to short-read sequencing data [50, 51], which may result in missing true positive pAs due to insufficient read coverage or introducing false positive sites due to alignment errors. To mitigate above issues, it is necessary to filter pAs obtained from ONT data to enhance their reliability, which inevitably leads to the loss of true pAs and thus underestimates the sensitivity of certain methods in detecting true positive sites. Previously, Zhao *et al*. [34] selected pAs in common genes identified in the ONT data and across all methods for assessment, which led to the inability to assess the authenticity of pAs on non-consensus genes and might cause deviations in the performance evaluation of certain methods. Due to the lack of matched ONT datasets across diverse scRNA-seq samples and the inherent technical limitations of ONT data, we instead collected extensive pA annotations from bulk 3′ seq data to serve as the ground-truth reference (Fig. 1a). These annotations cover most known pAs across different species and tissues, enabling the assessment of prediction accuracy for 3′ UTR pAs across various methods. Considering that existing pA annotations do not cover all real sites from biological samples, especially tissue-specific sites, we separately evaluated the unique pAs identified by each method that were not present in the pA annotations (Fig. 5c–e), providing a more comprehensive evaluation of the pA identification performance of each method. Our results showed that annotation-based methods, Infernape excepted, displayed high localization precision, yet their requirement for pre-compiled pA annotations restricts their use to species beyond human and mouse, where annotation is incomplete. MAAPER, in particular, refines every predicted site to the nearest annotated coordinate; this forced alignment yields superior scores against reference benchmarks on both simulated and real datasets (Figs 2a and b, 3d and g, and 4a and b), but risks inflating apparent performance and systematically overlooks genuine novel pAs absent from the annotation. Among the peak calling-based methods used for *de novo* pA identification, polyApipe outperformed others and maintained stable performance across both simulated and real datasets, further validating its reliability. Although within a 50 nt window size, a large number of pAs identified by SCAPE fell into the overlapping regions of reference pAs, its false positives were also relatively high (Fig. 4a). Further examination of the poly(A) signal motifs and single nucleotide profiles revealed that the identified sites by SCAPE and scAPA showed significant positional offsets relative to the true sites (Fig. 3e–g). It should be noted that methods such as SCAPE and Infernape report only a poly(A) peak range rather than an exact pA coordinate; therefore, we first reduced each range to a single representative position and treated it as the coordinate of the identified pA before benchmarking accuracy. Concretely, we adopted the mean-position rule from the original SCAPE paper [22], whereas for Infernape, scAPA and related callers we assigned the 3′-most base, following respective publications [19, 22, 27]. This process inevitably introduces a positional error for some methods, because ranges returned by model-driven quantifiers such as SCAPE and Infernape are optimized for abundance estimation rather than nucleotide-level localization, which partly explains their lower precision in our pA-identification evaluation.

In terms of pA quantification performance, we evaluated pA expression profiles obtained by different methods at both the gene and pA levels, and further assessed pA quantification performance by calculating DEAPA genes. First, evaluations on simulated datasets showed that SCAPTURE and SCAPE outperformed all other methods in pA quantification at both the barcode and group levels. They exhibited significantly lower MAPE values and maintained consistently low errors across datasets from different species, demonstrating strong robustness (Fig. 2c and d). This observation was consistent with the results of our subsequent evaluations on real datasets. We observed significant differences between the gene expression profiles obtained from APA analysis methods and the results from gene quantification methods across real datasets from different sequencing protocols. Based on 10X Chromium and Microwell-seq data, most methods showed relatively high correlations (Fig. 6a). However, correlations were generally lower for CEL-seq data, with only SCAPE and SCAPTURE performing well. In the correlation analysis of expression of consensus pAs between different methods, compared with other sequencing datasets, most methods also showed significantly lower correlation for CEL-seq data. This difference may be related to the low coverage, high noise level, and sequencing platform characteristics of CEL-seq data. Therefore, when performing APA analysis with CEL-seq data, particular attention should be paid to selecting appropriate methods and interpreting results with caution. For instance, considering both identification and quantification results, SCAPTURE may be more suitable for CEL-seq data. We referred to previous studies [20, 25, 52] and used matched 3′ seq and bulk RNA-seq data as ground truth to validate the accuracy of different methods in pA quantification. We observed that SCAPE, scUTRquant, SCAPTURE, and polyApipe showed stronger correlations with the ground truth for APA quantification (Fig. 6f). For the identification of DEAPA genes, the evaluation using real data is challenging due to the lack of reference DEAPA genes for most real datasets and the lack of matched bulk 3′ seq data. To address this challenge, we proposed a strategy for constructing a consensus set of DEAPA genes using global DEAPA gene detection results as a reference and compared it with the differential analysis results from two 3′ seq datasets to verify the reliability of the consensus set. Additionally, we confirmed the biological rationality of these genes through GO analysis (Fig. 7d). These strategies for evaluating DEAPA provide practical guidance for future studies to evaluate the pA quantification results.

Surprisingly, we found that the accuracy of positions of identified pAs does not seem to be closely related to the results of pA quantification. This may be because the expression level of a pA is typically based on the sequence coverage in the vicinity (i.e. peak) of a pA, hence pA quantification has a certain tolerance for deviations in the pA position. For example, SCAPE exhibits lower precision for pA identification, but demonstrates higher accuracy and robustness for pA quantification across different sequencing protocols. SCAPE used a probabilistic mixture model to infer pAs, modeling pAs as a Gaussian distribution with mean *α* and standard deviation *β*. When the predicted pA site has some deviation, as long as this deviation does not exceed the reasonable range described by the uncertainty *β*, the model can still capture most reads belonging to this site through the probability distribution. Second, in SCAPE, for reads falling in ambiguous regions between two predicted pAs, a probability allocation mechanism calculates the probability of the read belonging to different pAs. These strategies can mitigate positional offsets to some extent, thereby allowing for a relatively reliable quantification of pAs. Therefore, in practical applications, appropriate methods should be selected based on the research purpose—pA identification or pA quantification.

There are some limitations of our study. Regarding species coverage, our inclusion of *Arabidopsis* alongside human and mouse addresses known biological differences [e.g. poly(A) signals and microheterogeneity [47]] between plants and animals, providing a more comprehensive cross-kingdom evaluation than previous studies. However, we only focused on these three models due to insufficient high-quality pA annotations and/or scRNA-seq data for other species. In the future, our open pipeline will enable quick extension of these benchmarks to new species as data becomes available, further enhancing the generalizability of our results. Second, the lack of ONT data from comprehensive tissue samples across different species restricts the use of ONT data for APA analysis of single-cell transcriptomes [53, 54]. We compiled prior pA annotations from bulk 3′ seq as the ground truth for pA identification assessment. However, this annotation-dependent strategy risks overlooking *bona fide* APA events and may artificially inflate the apparent accuracy of tools—such as MAAPER—that exploit given annotations to refine pA positions. Meanwhile, using 3′ seq and bulk RNA-seq data as ground truth inevitably fails to consider cell heterogeneity and cannot fully evaluate identification results at the single-cell level. Considering the advantages of ONT in identifying transcript structure, it is suggested that in the future, the identification and quantitative results of 3′ seq and ONT data can be combined as a more comprehensive reference. Furthermore, due to the lack of matched 3′ seq data for diverse scRNA-seq protocols, we only evaluated the quantitative results of different methods on the 10X Chromium dataset, while the quantitative performance on other sequencing data remains unknown. Moreover, most current tools—including roughly half of those benchmarked here—restrict their search to 3′ UTR pAs, so intronic pAs are either ignored or reported at very low numbers. Particularly, intronic pAs identified from scRNA-seq data are intrinsically noisy: low abundance, prevalent internal-priming artefacts, and the lack of orthogonal assays render reliable validation elusive [20, 26]. For these reasons we limited the present evaluation to 3′ UTR pAs. Yet intronic cleavage sites can flag retained introns within unspliced pre-mRNAs, a regulatory state that is increasingly implicated in cell-type-specific physiology; developing robust, experimentally validated algorithms for intronic-pA discovery therefore remains a critical next step.

## Supplementary Material

gkag490_Supplemental_Files

## Data Availability

Sequencing datasets used in this study are summarized in Supplementary Table 2 and can be publicly accessed via the accession numbers provided in the table. The reference pA datasets and code involved in this study are available at https://github.com/BMILAB/Benchmark_scAPA, licensed under the MIT license, and Zenodo at https://zenodo.org/records/19903047 DOI: 10.5281/zenodo.19903047.
