# Supplementary material for: Benchmarking computational methods for identifying and quantifying polyadenylation sites from 3′ tag-based single-cell RNA-seq data
Source: Nucleic Acids Res. 2026 May 12;54(9):gkag490. doi: 10.1093/nar/gkag490 (PMC13161560; doi:10.1093/nar/gkag490)
Supplement: gkag490_Supplemental_Files [file gkag490_supplemental_files.zip › Supplementary Figures.pdf]

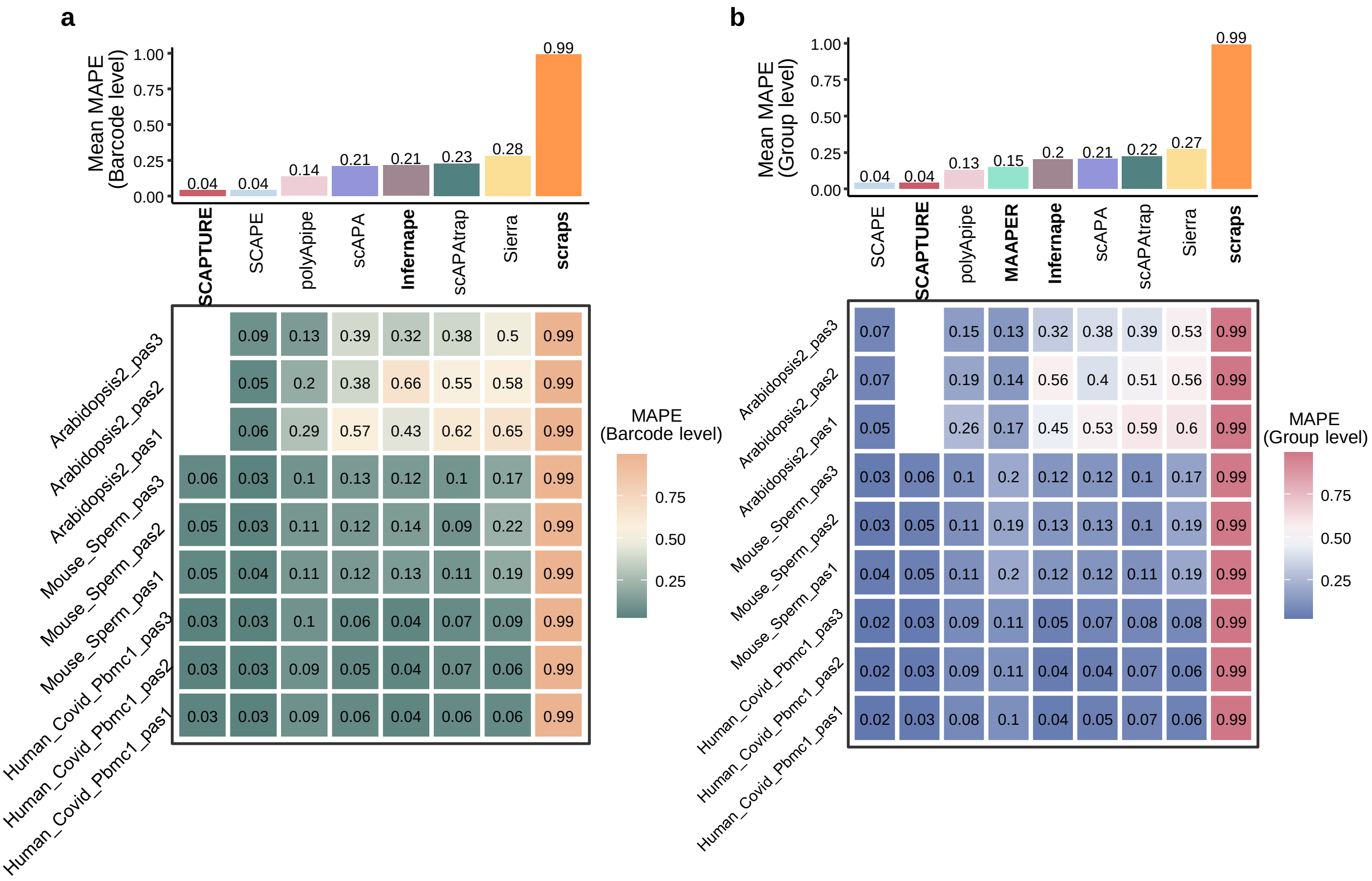


**Supplementary Figure 1. Evaluation of methods for pA quantification of commonly identified pAs using simulated scRNA-seq datasets. a.** Mean absolute percentage error (MAPE) for pA quantification at the barcode level across different datasets. Only the pAs that matched the ground truth and were commonly identified by at least six methods were included in the calculation. Lower MAPE values indicate higher quantification accuracy. Since MAAPER only generated the total expression level of pAs in a cell population, it was not included in the MAPE calculation at the barcode level. **b.** Same as a, but showing MAPE at the group level.

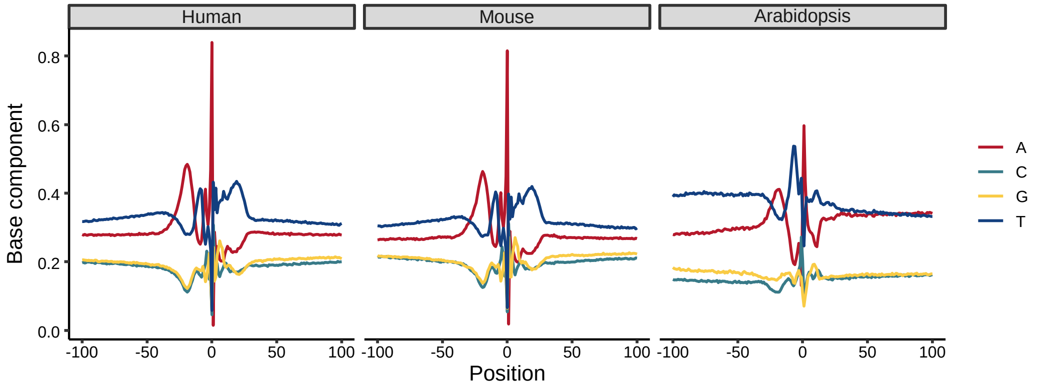


**Supplementary Figure 2. Single nucleotide profiles of reference 3' UTR pAs from different species.** Y-axis denotes the fractional nucleotide content at each position. X-axis denotes the position and 0 is the position of the pA.

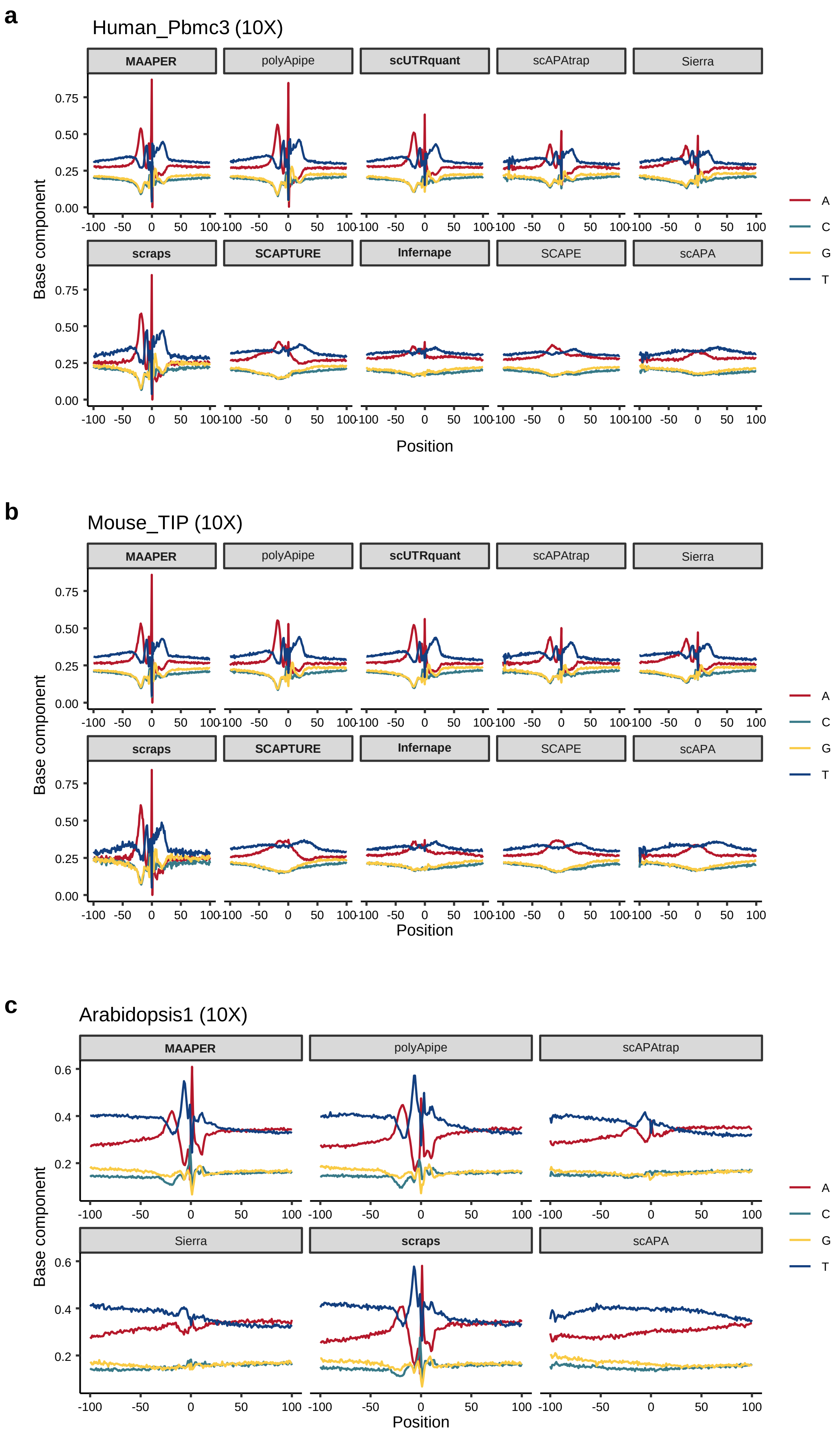
a
Human_Pbmc3 (10X)
MAAPER
polyApipe
scUTRquant
scAPAtrap
Sierra
scraps
SCAPTURE
Infernape
SCAPE
scAPA
Base component
0.00
0.25
0.50
0.75
-100
-50
0
50
100
Position
A
C
G
T
b
Mouse_TIP (10X)
MAAPER
polyApipe
scUTRquant
scAPAtrap
Sierra
scraps
SCAPTURE
Infernape
SCAPE
scAPA
Base component
Position
A
C
G
T
c
Arabidopsis1 (10X)
MAAPER
polyApipe
scAPAtrap
Sierra
scraps
scAPA
Base component
0.2
0.4
0.6
Position
A
C
G
T

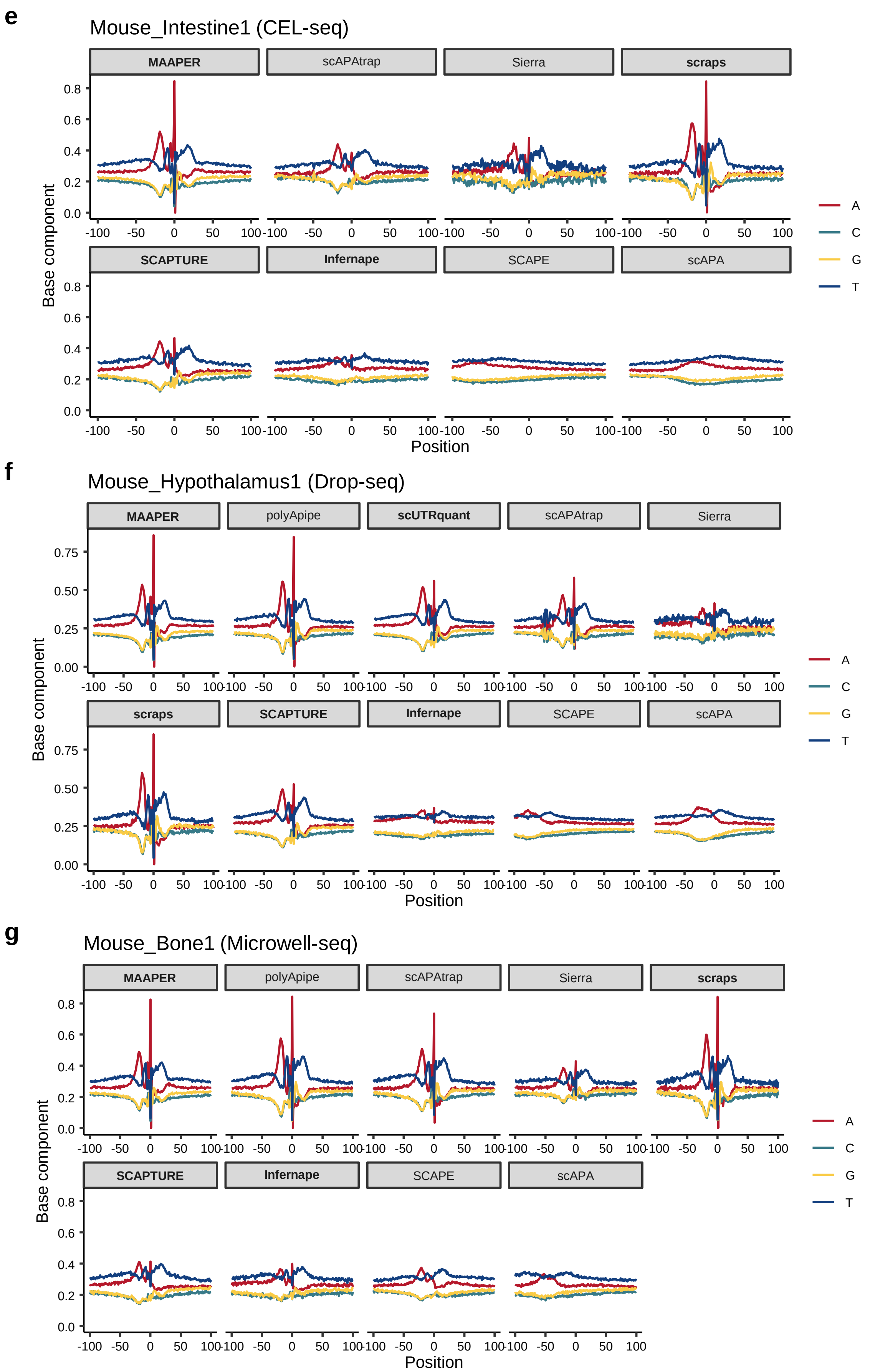


**Supplementary Figure 3. Nucleotide compositions of the sequences surrounding 3′ UTR pAs identified by each method across datasets from different sequencing protocols. a-g.** Single nucleotide profiles of 3’ UTR pAs in six representative datasets from different species and sequencing protocols. Y-axis denotes the fractional nucleotide content at each position. X-axis denotes the position and 0 is the position of the pA.

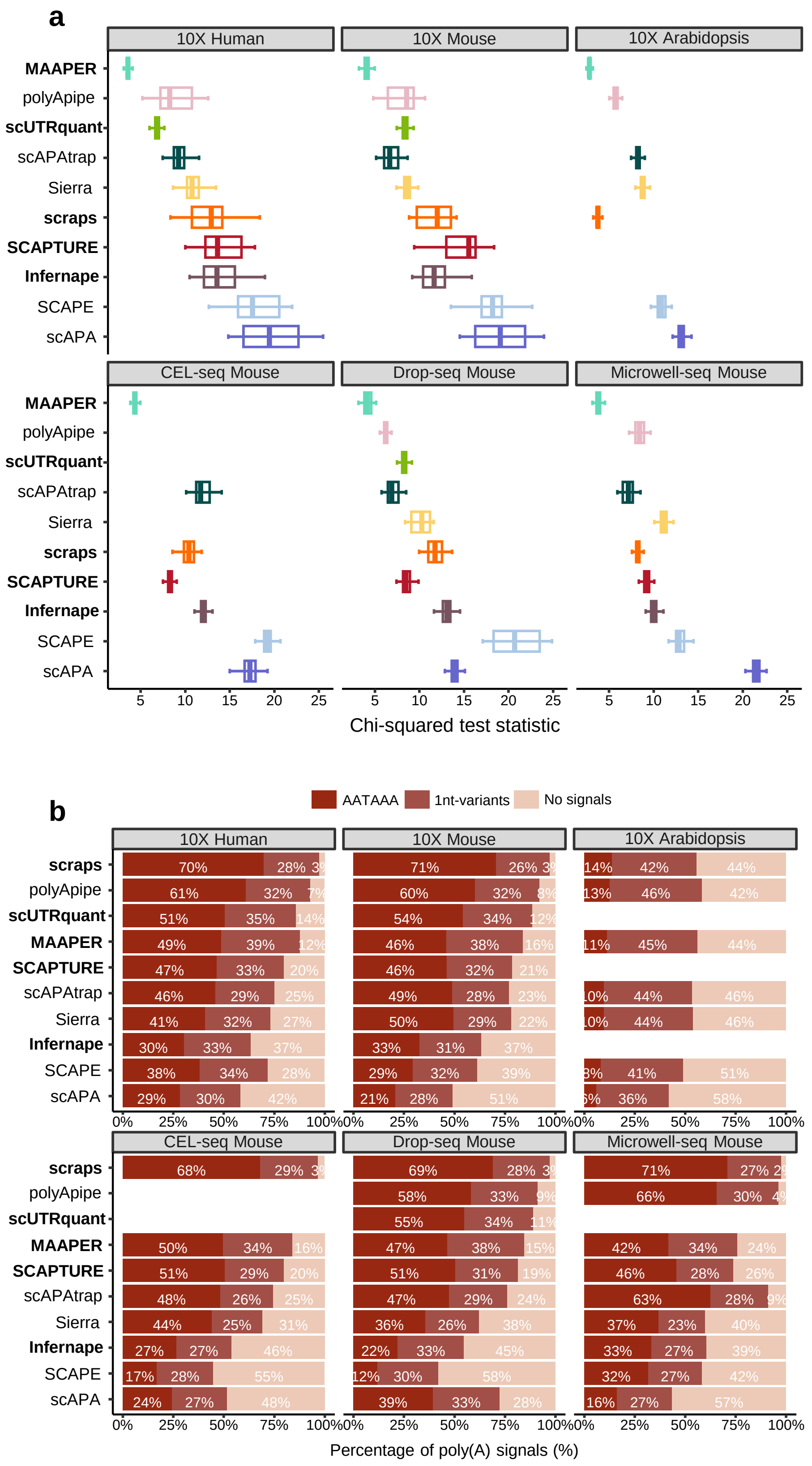


**Supplementary Figure 4. Analysis of sequence characteristics of 3' UTR pAs identified by each method across different sequencing datasets. a.** Boxplots showing scores of chi-squared metric of 3' UTR pAs identified by each method across datasets from different sequencing protocols. The smaller the chi-square value, the more consistent the single nucleotide profile between the identified pAs and the reference ones (Supplementary Fig. 2). **b**. Average proportion of 3' UTR pAs containing polyA signal motifs identified by each method for datasets from different sequencing protocols.

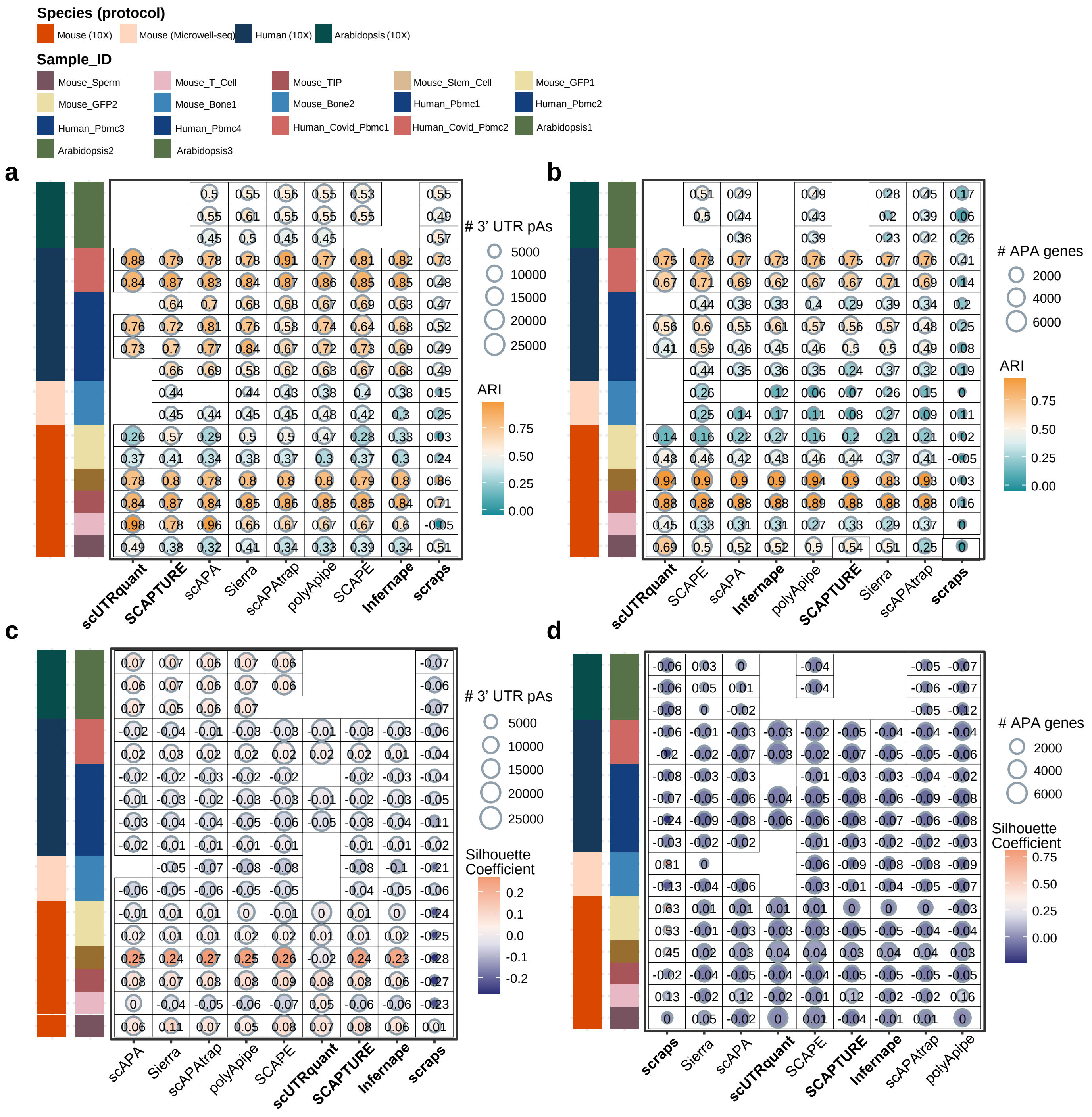

**Supplementary Figure 5. Evaluation of pA quantification across different methods based on cell clustering. a.** ARI scores calculated based on pA expression profiles across 17 datasets from 10X Chromium and Microwell-seq. **b.** Same as a, except that ARI scores were calculated based on APA usages measured by relative usage of the distal pAs (RUD). **c.** Same as a, except that SC scores were calculated based on pA expression profiles. **d.** Same as a, except that SC scores were calculated based on RUD.

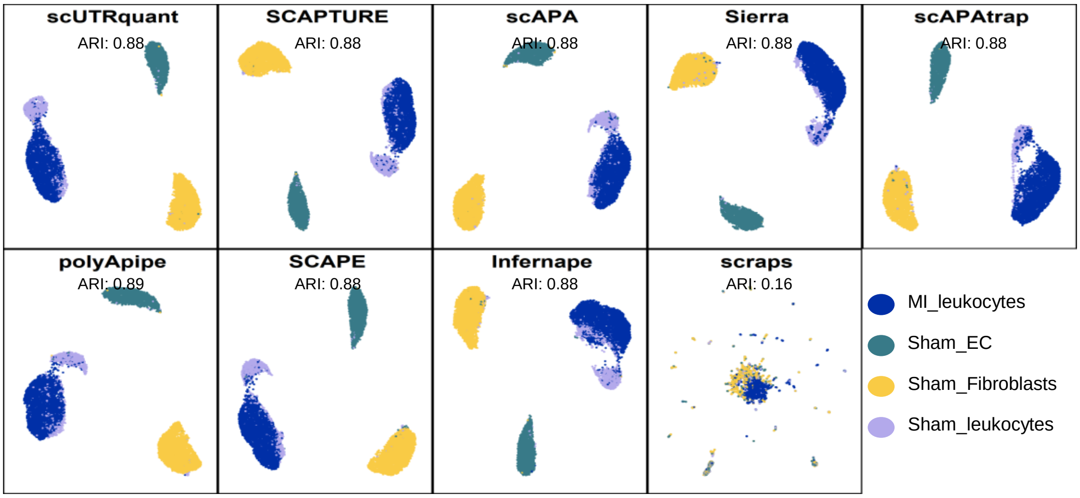


**Supplementary Figure 6. UMAP plot showing the cell clustering results for the mouse TIP data based on the RUD (Relative Usage of the Distal pA) profile obtained from each method.**

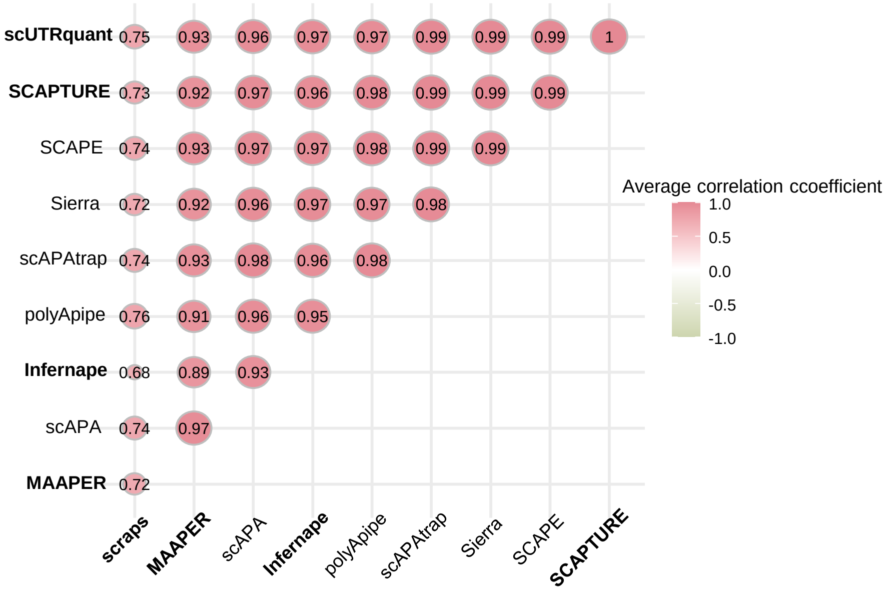


**Supplementary Figure 7. Pearson correlation coefficients between pA expression profiles of consensus pAs quantified by different methods.** Consensus pAs are those commonly identified by all the ten methods. Only datasets with > 200 consensus pAs were used, *i.e.*, 10X datasets of mouse and human.

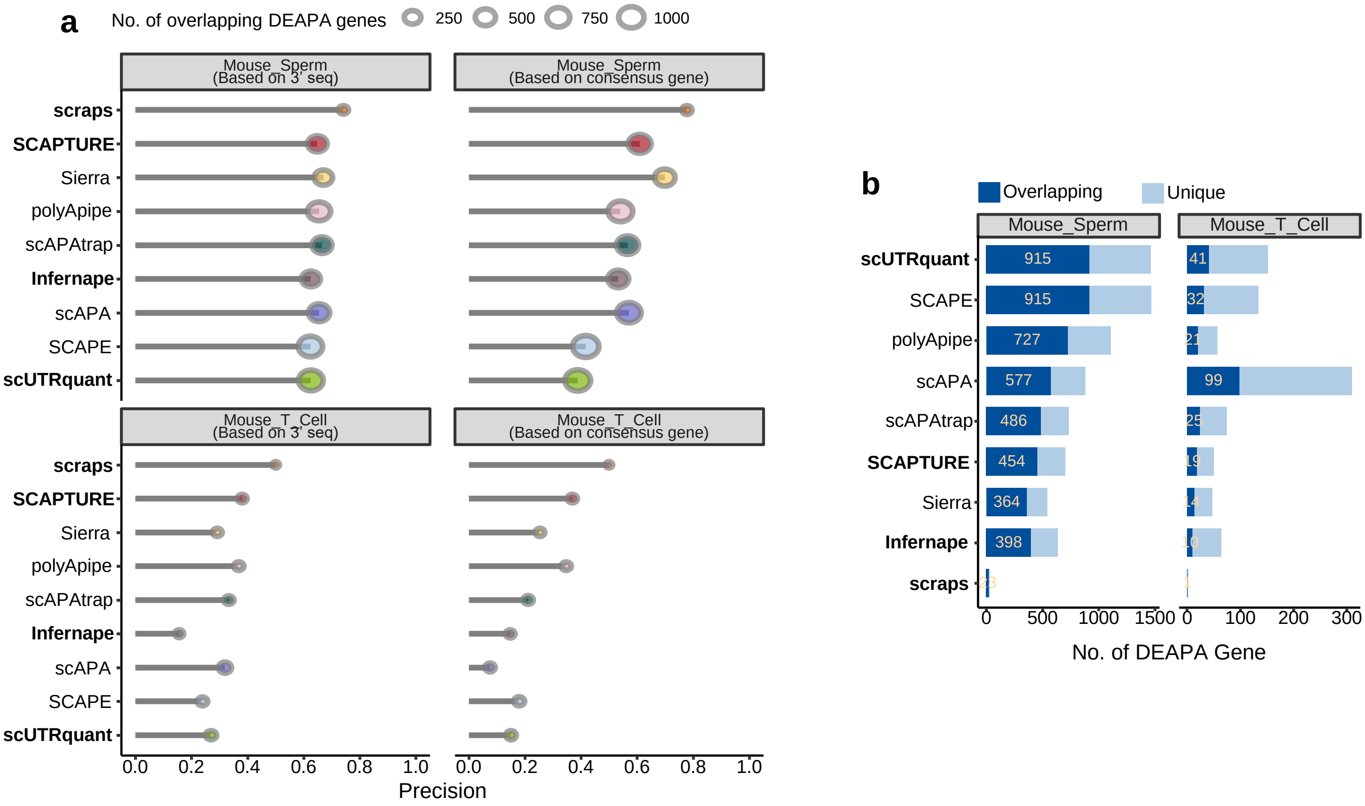


**Supplementary Figure 8. Performance evaluation of methods in identifying DEAPA genes using DEAPA genes from 3′ seq datasets as the reference. a.** The precision of DEAPA genes detected by each tool was calculated using the consensus DEAPA gene set (right) and DEAPA genes based on 3′ seq data (left) as the ground truth, respectively. **b.** Number of DEAPA genes detected by each method for each scRNA-seq dataset. Overlapping DEAPA genes are defined as those present in the DEAPA gene set identified from the matched 3′ seq dataset. Unique DEAPA genes are those only identified in the scRNA-seq data.

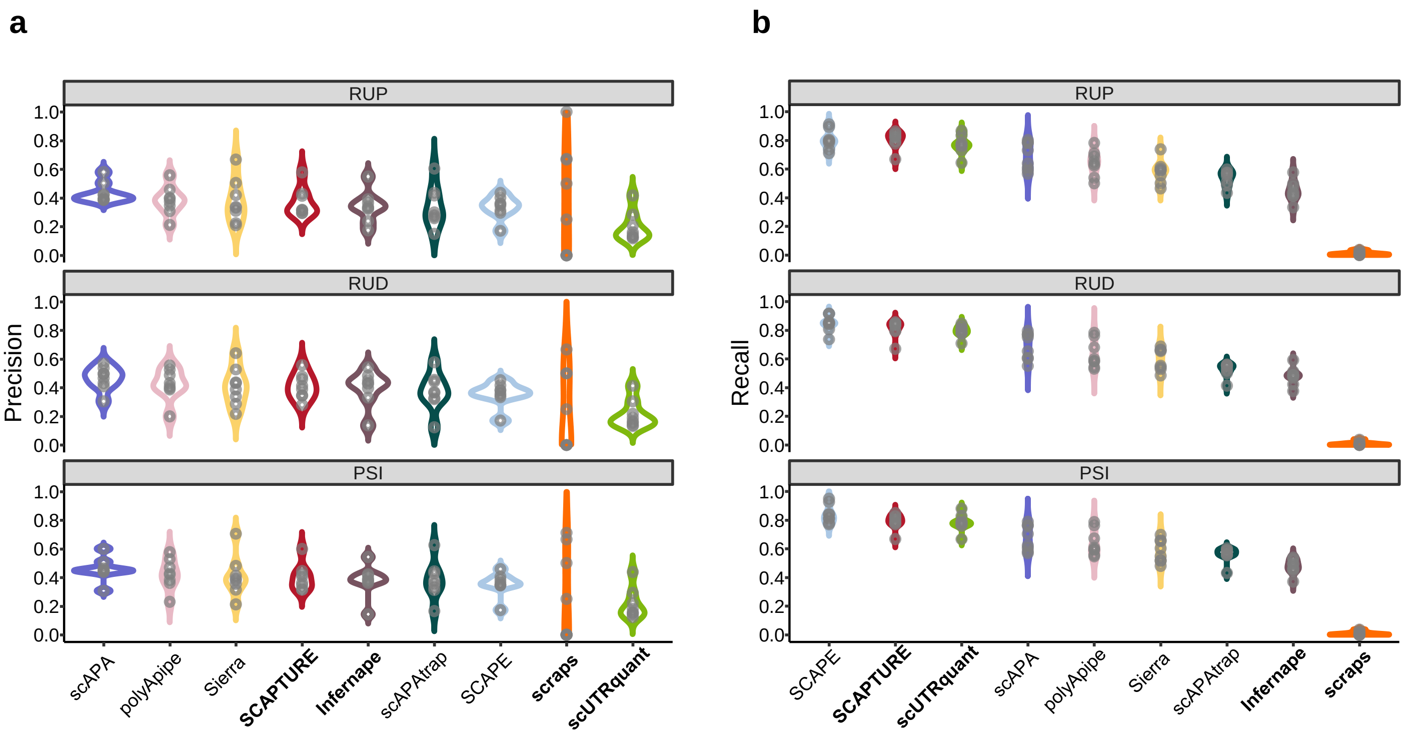


**Supplementary Figure 9. Performance evaluation of methods in identifying DEAPA genes using Wilcoxon rank-sum test. a.** Precision of DEAPA genes identified by each method calculated using the consensus DEAPA gene set as the ground truth. DEAPA genes were detected by applying the Wilcoxon rank-sum test on three distinct APA usage indices separately: RUP (Relative Usage of the Proximal site), RUD (Relative Usage of the Distal site), and PSI ($\psi$). **b.** Performance metrics as in panel a, but showing the recall of DEAPA genes detected by each method.
