# Supplementary material for: Benchmarking computational methods for identifying and quantifying polyadenylation sites from 3′ tag-based single-cell RNA-seq data
Source: Nucleic Acids Res. 2026 May 12;54(9):gkag490. doi: 10.1093/nar/gkag490 (PMC13161560; doi:10.1093/nar/gkag490)
Supplement: gkag490_Supplemental_Files [file gkag490_supplemental_files.zip › Supplementary Text.pdf]

Benchmarking computational methods for identifying and quantifying polyadenylation sites from 3′ tag-based single-cell RNA-seq data

Supplementary Text

MATERIALS AND METHODS 2
Data collection and preprocessing 2
Existing computational methods for pA identification and quantification 2
Annotation of pAs 5
Collection of the reference pA datasets 5
Benchmarking on simulated data 6
Data simulation 6
Evaluation of pA quantification on simulated data 6
Evaluation of pA identification 7
Evaluation of unique pAs..8
Evaluation of pA quantification 9
Evaluation of pA quantification at the gene level 9
Evaluation of pA quantification at the pA level 11
Evaluation of DEAPA gene identification 12
Datasets for DEAPA evaluation 12
Construction of the consensus DEAPA gene set 12
Metrics for evaluating DEAPA gene identification 14

MATERIALS AND METHODS

Data collection and preprocessing

To ensure a comprehensive and representative evaluation, we systematically compiled publicly available datasets (Supplementary Table 2) that had previously served as benchmarks for the methods examined in this study. Specifically, we collected 25 public datasets spanning four scRNA-seq protocols, including 15 10X Chromium, six CEL-seq, two Drop-seq, and two Microwell-seq datasets (Supplementary Table 2). These datasets span multiple species and sequencing protocols, capturing a broad spectrum of biological and technical variation to more rigorously evaluate each method's accuracy and generalizability across diverse conditions. Before alignment, we used FastQC to assess the quality of the sequencing data and confirmed that these sequencing datasets did not contain sequencing adapters. Next, the fastq files for 10X Chromium were aligned using Cell Ranger (v3.1.0), those for CEL-seq and Drop-seq were aligned using STARsolo, and the fastq files for Microwell-seq were processed according to the tutorial at the GitHub repository (https://github.com/zhou-ran/workflows/tree/master/DropSeq_processing). Finally, all reads from human, mouse, and *Arabidopsis* data were aligned to the GENCODE v34 hg38 (1), GENCODE v25 mm10 (1), and Ensembl TAIR10.49 reference genomes, respectively. Moreover, we used SAMtools to extract alignment information for high-quality cells filtered based on gene expression and annotated with cell type labels from BAM files. The same filtered BAM files were supplied to every method to guarantee an identical starting point.

In addition, we collected 3′ seq data matched with scRNA-seq data from mouse sperm and mouse T cells, as well as bulk RNA-seq data matched with mouse TIP scRNA-seq data (Supplementary Table 2), to evaluate the accuracy of different APA analysis methods for pA quantification and DEAPA gene recognition.

Existing computational methods for pA identification and quantification

This study benchmarked ten methods (Supplementary Table 1), which can be grouped into two categories.

i. *De novo* pA identification methods, including scAPA, polyApipe, Sierra, scAPAtrap, and SCAPE. scAPA (2) uses Homer (3) to detect pAs by peak calling and uses mclust (4) to separate overlapping peaks based on a Gaussian mixture model. Additionally, peaks having a genomic sequence of at least eight consecutive As in the region between 10 nt and 140 nt downstream of their 3′ edge are suspected to result from internal priming and can optionally be excluded from the analysis.

polyApipe (https://github.com/MonashBioinformaticsPlatform/polyApipe) identifies potential pAs by detecting soft-clipped reads with poly(A) and assigns additional reads to these sites if they align in the upstream 250 bp. Subsequently, the algorithm flags presumed internal priming sites based on the presence of arbitrarily defined genomic poly(A) homopolymers. polyApipe is employed within the PASTA module (5) for pA identification and quantification; PASTA interfaces directly with the widely used Seurat framework.

Sierra (6) uses splice-aware peak calling based on Gaussian curve fitting to determine potential peaks with pAs. If genomic sequences and annotations are provided, the annotation step will determine whether the peaks overlap with exons, introns, or UTRs, and whether they may be derived from A- or T-rich regions.

scAPAtrap (7) incorporates peak calling strategy and poly(A) read anchoring, which can accurately locate pAs without using prior genome annotation. The peak calling step identifies potential regions where pAs are located, and the pA anchoring step extracts reads with A/T stretches and determines precise locations of pAs.

SCAPE (8), a Bayesian method, enables *de novo* identification of pAs by utilizing insert size information. To avoid capturing internal priming pAs, each candidate pA is compared with the internal priming regions and is removed when this pA is proximal to these priming regions.

ii. Methods based on prior pA annotations, including MAAPER, SCAPTURE, Infernape, scUTRquant, and scraps.

MAAPER (9) is a model-based method that identifies pAs by analyzing the distances of 3′ end-linked reads to true pAs. At the pA prediction stage, MAAPER first uses genes that contain only a single pA from PolyA_DB3 (10) to learn the distribution of read-pA distance. Then, based on the

learned read-pA relationships, it uses a likelihood model to predict the pA for each 3′ end-linked read mapped to a given gene.

SCAPTURE (11) considers raw peaks containing pAs from scRNA-seq read signals and embeds the deep learning model DeepPASS. This model uses a sequence shifting strategy around known pAs to further select high-confidence pAs.

Infernape (12) first detects raw peak modes, and then, based on the approach used in scAPA and Sierra, it applies a local parametric Gaussian density model of the read counts to refine the peak mode locations and their spread. It also uses established pA annotations to filter out peaks that are unlikely to correspond to authentic pAs.

scUTRquant (13) is a pipeline for recovering 3′ UTR isoform counts from 3′ tag-based scRNA-seq datasets, and it quantifies the expression of 3′ UTR isoforms by integrating cleavage site annotations from GENCODE (1) annotations and the Microwell-seq dataset.

scraps (14) is designed for identifying pAs at near-nucleotide resolution in single cells using 10X Genomics and other TVN-primed scRNA-seq data. For paired-end alignment, scraps uses the positional information encoded in read 1 of common TVN-primed libraries to locate pA; for read 2-only alignment, scraps locates pAs by identifying soft-clipped reads containing poly(A) sequence. In addition, scraps uses known pAs to distinguish between true pAs and false positive pAs resulting from internal priming.

We ran each method following the respective tutorials and default parameters. Since different methods are applicable to different species and sequencing protocol data, certain methods did not run successfully on specific data (Supplementary Table 3). For methods requiring reference pAs to assist prediction for *Arabidopsis* data, we provided our own collected *Arabidopsis* reference pAs. Among these methods, the DeepPASS model built in SCAPTURE does not currently support pAs prediction for *Arabidopsis* data; Infernape failed to obtain expression counts for pAs in single cells using default parameters; scUTRquant requires a pre-built *Arabidopsis* reference target transcriptome. Consequently, these methods did not run successfully on *Arabidopsis* datasets.

Annotation of pAs

Firstly, we uniformly filtered out low expression pAs, retaining only those expressed in at least 0.5% of cells. To filter out pAs misidentified due to internal priming, we used the strategy provided by each method to analyze whether pAs originated from A- or T-rich regions. Next, for methods directly outputting precise coordinates of pAs, we annotated the genomic regions of pAs based on their coordinates. For methods that output only a poly(A) peak range, we first collapsed the interval to a single representative coordinate before accuracy assessment: the mean position for SCAPE (8) and the 3′-most base for Infernape, scAPA and other related callers, mirroring the conventions established in their respective publications (2,8,12). Moreover, to minimize errors due to different annotation methods, we uniformly used the annotatePAC function provided in the movAPA package (15) to annotate pAs. To recruit pAs in the downstream region adjacent to the 3′ end, the annotated 3′ UTRs were extended by 2000 bp, 2000 bp, and 1000 bp for human, mouse, and *Arabidopsis*, respectively. Since scAPA was developed with its own built-in GENCODE reference (v33 hg19) (1), genomic intervals of scAPA-called peaks were transformed to the hg38 reference genome using UCSC LiftOver before annotating pAs.

Collection of the reference pA datasets

We collected prior pA annotations as ground truth for evaluating pA identification results. First, we obtained human and mouse pA annotations from GENCODE (v44 and vM33) (1) and polyA_DB 3 (10). Since the human and mouse pA annotations collected from polyA_DB 3 were constructed based on the hg19 and mm9 reference genomes, we used UCSC LiftOver to transfer the genomic coordinates to the hg38 and mm10 reference genomes. We obtained *Arabidopsis* pA annotations from previous studies (16,17). Following the previous study (18), we created the reference pA dataset by incorporating pA annotations from diverse sources of the same species. To facilitate comparison of the distance distributions between identified pAs and reference pAs, we unified the reference genomes to human hg38, mouse mm10, and *Arabidopsis* TAIR10, respectively. To obtain 3′ UTR reference pAs, we annotated pAs and extended annotated 3′ UTRs using movAPA (15).

Ultimately, we obtained 117,674 human pAs, 132,514 mouse pAs, and 41,267 *Arabidopsis* pAs (Fig. 1a).

Benchmarking on simulated data

Data simulation

To evaluate the performance of APA detection methods, we generated 3' tag-based scRNA-seq data with known APA events while preserving partial peak characteristics from real data, following the simulation scheme proposed by Li *et al.* (19) (code is available at https://github.com/Lycidas97/apabenchmark). Based on BAM files from human peripheral blood mononuclear cells (GSM4712885), mouse sperm cells (GSE140556), and *Arabidopsis* root cells (GSM3490690), we generated simulated data across three species, with three replicates for each type. Notably, to extract non-overlapping peaks near genuine pAs from BAM files for subsequent peak feature construction in simulated data, we selected pAs with no other pAs within 500 bp upstream or downstream from human and mouse pA annotations. Due to narrower pA intervals, we selected pAs with no other pAs within 200 bp upstream or downstream from *Arabidopsis* pA annotation. Additionally, to define genuine APA events in simulated data, for genes with multiple pAs, we selected pAs that were at least 200 bp apart from each other within the same gene in human and mouse pA annotations, and at least 100 bp apart in *Arabidopsis* pA annotation.

Due to the failure of scUTRquant in processing simulated BAM files, the benchmarking on simulated data included only nine methods.

Evaluation of pA quantification on simulated data

We evaluated the pA quantification accuracy of each method by calculating the MAPE between the pA expression matrix and the ground truth expression matrix at both barcode and group levels. Predicted pAs within a 24 nt window of ground truth sites were considered matched and included in the calculation. When a predicted pA matched multiple ground truth sites, it was assigned to the nearest ground truth site; when a ground truth site matched multiple predicted pAs, the sum of the

expression values of these predicted pAs was used as the predicted expression value of that ground truth site. The MAPE is defined as follows:

$$MAPE_{barcode} = \frac{1}{n \times m} \sum_{j=1}^{n} \sum_{i=1}^{m} \frac{|c_{i,j} - \hat{c}_{i,j}|}{c_{i,j}} \quad (1)$$

Here *n* is the number of barcodes, and *m* is the number of matched pAs (only those with non-zero ground truth expression are included). The absolute percentage error for pA *i* in barcode *j* is calculated as $\frac{|c_{i,j}-\hat{c}_{i,j}|}{c_{i,j}}$, where $c_{i,j}$ is the ground truth expression value and $\hat{c}_{i,j}$ is the predicted expression value.At the group level, MAPE was calculated based on the total expression of each pA across all cells.

$$MAPE_{group} = \frac{1}{m} \sum_{i=1}^{m} \frac{|c_i^{total} - \hat{c}_i^{total}|}{c_i^{total}} \quad (2)$$

Here *m* is the number of matched pAs (only those with non-zero ground truth total expression are included). The absolute percentage error for pA *i* across all cells is calculated as $\frac{|c_i^{total}-\hat{c}_i^{total}|}{c_i^{total}}$, where c_i^{total} is the ground truth total expression value and $\hat{c}_i^{total}$ is the predicted total expression value.

Evaluation of pA identification

To assess the sensitivity of pA identification, we calculated the pA set size score. Specifically, in each dataset, the score for the method identifying the most 3′ UTR pAs was assigned a value of 1, the score for the method identifying the least 3′ UTR pAs was assigned a value of 0, and the scores for other methods were normalized to the range of 0–1 using the (max – min) normalization method based on the number of pAs they identified.

To assess the accuracy of pA identification, we calculated the distance between identified 3′ UTR pAs and the nearest reference pAs. If an identified pA falls within the window size (0 to 100 nt) of any nearest reference pA, it is considered a true pA. Then the precision can be calculated (Eq. 3)

$$Precision_{pA} = \frac{TP_{pA}}{TP_{pA} + FP_{pA}} \quad (3)$$

TP_{pA} represents the number of identified pAs that match any reference pAs within a specified window size. FP_{pA} represents the number of identified pAs that do not match any reference pAs.

We proposed a chi-square metric to assess the similarity between identified pAs and reference pAs in terms of base compositions around pAs. For identified pAs and reference pAs, we calculated the frequency of four bases (A, T, C, and G) for each position in the upstream 100 nt to downstream 100 nt region of the pAs, and then used chi-square test (*chisq.test* function in R) to calculate the chi-square statistic for each position. Finally, the average chi-square statistic of all positions is taken as the measure, with smaller values indicating closer similarity to the reference profile. Specifically, we first randomly sampled 1,500 sequences which span 100 nt upstream and downstream of 3′ UTR pAs from the reference dataset, and calculated the frequency of four bases at different positions. We calculated their chi-squared metrics relative to the reference set for the selected sequences. This process was repeated 100 times. Ultimately, the 1,500 sequences with the smallest chi-squared value were chosen as the final reference dataset. Next, for each 3′ UTR pA dataset identified by different methods, we also randomly sampled 1,500 non-overlapping sequences. We calculated the frequency of four bases at different positions and compared these results with those obtained from the 1,500 reference sequences using a chi-square test. We repeated sampling 100 times, and a chi-square statistic can be obtained each time to represent the similarity between the nucleotide profile of the identified pAs and the reference pAs. If a method identified fewer than 1500 pAs in a given dataset, it would be excluded from further analysis.

To quantify how sequencing depth influences pA detection, we used samtools to down-sample the 146-million-read mouse-sperm BAM to 10%, 30%, 50%, 70% and 90% read fractions, reran each method on the reduced files, annotated the resulting 3′ UTR pAs, and compared the depth-dependent yield across methods.

Evaluation of unique pAs

To compare the consistency of identification results from the ten methods across different sequencing datasets, pAs identified from all methods were pooled together and then consensus pAs were obtained by grouping nearby sites within the 24 nt of each other. The agreement score (20) was used to assess the consistency of pA identification across different methods within a given dataset (Eq. 4).

$$\text{Agreement} = \frac{1}{|S|} \sum_{pAi \in S} \frac{n_{pAi} - 1}{N - 1} \tag{4}$$

Here N denotes the number of pA identification methods, S denotes the total number of pAs, consisting of all pAs identified by all methods, and n_{pAi} is the number of methods identifying pA *i*. The metric ranges from 0 to 1, where a value of 0 means that pA sets from all methods are completely distinct, and a value of 1 means that pA sets from all methods are the same.

Unique pAs of a method are defined as those sites not identified by any other method and absent from the reference pA dataset. To validate the authenticity of identified unique pAs, we chose the DeepPASTA model (21). At the time of its release, DeepPASTA outperformed other contemporary deep-learning models for pA prediction, and a recent benchmark (11) confirmed that it remains among the top-performing tools despite the emergence of newer architectures; this sustained accuracy makes it a reliable choice for cross-validating our candidate sites. We used DeepPASTA to predict unique pAs identified by each method in human and mouse data based on the 200 nt sequences surrounding the pAs and the RNA secondary structure data. A predicted probability ranging between 0 and 1 of a given pA is obtained by DeepPASTA, resulting in its classification as a positive site (predicted likelihood values > 0.5) or a negative site (predicted likelihood values ≤ 0.5). Finally, we estimated the proportion of unique pAs identified by each method that were predicted as positive by DeepPASTA.

Evaluation of pA quantification

Evaluation of pA quantification at the gene level

To compare gene-level quantification results from different methods against those from gene quantification methods, we summed the expression counts of all pAs for each gene to obtain gene-level expression counts. For 10X Chromium data, we used Cell Ranger for gene quantification; for Microwell-seq data, we used STAR; and for CEL-seq and Drop-seq data, we used STARsolo. Subsequently, we calculated the Pearson correlation coefficient between the gene expression obtained from different methods and the results from gene quantification methods, considering only common genes.

We also performed cell type clustering to evaluate the quantification results. We used 17 datasets with cell type annotations (Supplementary Table 2). We compared clustering performance based on gene expression counts, pAs expression counts, and APA usages, respectively. The APA usage is quantified as RUD for each gene in each single cell (Eq. 5)

$$RUD_{s,i} = \frac{E_{d,i}}{\sum E_i} \tag{5}$$

The RUD for gene *i* in cell s is calculated as the ratio of the read counts for distal pA to the total read counts for all 3′ UTR pAs on that gene. The RUD score ranges between 0 and 1, with a higher RUD indicating increased use of the distal site, *i.e.* lengthening of the 3′ UTR.

We used the external metric ARI and the internal metric SC to assess clustering accuracy, respectively. As an external metric, ARI measures the similarity between two clusters by comparing clustering labels with true cell labels (Eq. 6).

$$\boldsymbol{RI} = \frac{\mathbf{a} + \mathbf{b}}{\boldsymbol{C}_N^2} \tag{6}$$

$$\boldsymbol{ARI} = \frac{\boldsymbol{RI} - \boldsymbol{E}\lfloor \boldsymbol{RI} \rfloor}{\boldsymbol{max}(\boldsymbol{RI}) - \boldsymbol{E}\lfloor \boldsymbol{RI} \rfloor}$$

The *RI* is the proportion of correctly classified sample pairs. *a* represents the number of sample pairs that belong to the same class in both the true labels and the clustering results. *b* represents the number of sample pairs that do not belong to the same class in both the true labels and the clustering results. C_N^2 is the total number of sample pairs. $E\lfloor RI \rfloor$ is the expected value of *RI* and $max(RI)$ is the maximum possible value of *RI*. The value of ARI ranges from -1 to 1, with value closer to 1 indicating higher clustering similarity.

As an internal metric, SC does not require cell annotation labels and assesses clustering results based on the closeness of each sample to the cluster to which it belongs and its separation from other clusters (Eq. 7).

$$SC = \frac{b(i) - a(i)}{max\big(a(i),\ b(i)\big)} \tag{7}$$

Here $a(i)$ represents the average distance between sample i and other samples within its own cluster, and $b(i)$ represents the average distance between sample i and samples in the nearest neighboring cluster. The value of SC also ranges from [-1, 1], with values closer to 1 indicating

better clustering performance, where cell samples are tightly grouped within their clusters and well-separated from others.

Notably, MAAPER was designed to quantify the total expression of pAs for cell populations rather than single cells, and was therefore excluded from the evaluation of cell clustering, PAU quantification, and DEAPA identification.

Evaluation of pA quantification at the pA level

To compare the consistency of different methods for pA quantification, we also calculated the Pearson correlation coefficients between the total expression counts of consensus pAs for each pair of methods. Consensus pAs were defined as pAs within 24 nt of each other. Moreover, we defined high-confidence pAs as pAs identified by all methods. We retained 12 datasets where the number of high-confidence pAs was greater than 200. For each dataset, we also calculated Pearson correlation coefficient for expression levels of high-confidence pAs between different methods.

Meanwhile, we used matched 3′ seq and bulk RNA-seq data as ground truth to assess the consistency of different methods for PAU quantification by comparing the differences between proximal and distal PAUs of the same gene. For 3′ seq data, if previous studies provided identified and quantified pA data (*i.e.*, mouse T cell data), we directly used these data; if not available (*i.e.*, mouse sperm cell data), we applied polyAseqTrap (22) to identify and quantify pAs. For bulk RNA-seq data (*i.e.*, mouse TIP data), we applied QAPA, which performed well in benchmarking of methods that identify and quantify pAs from RNA-seq data (23,24), to identify and quantify pAs. Subsequently, we used the *filterByExpr* function of edgeR (25) for filtering, setting the parameters *min.count* = 2 (when the pA expression matrix is tag per million data) or *min.count* = 5 (when the pA expression matrix is raw count data), *min.total.count* = 10, and *min.prop* = 0.8 to remove low-confidence pAs.

Given the significant differences in the number of APA genes identified by the nine methods during the evaluation, if only shared APA genes are used for evaluation, the accuracy of quantifying PAU for non-shared APA genes will be difficult to assess for methods that identify a large number of APA

genes. Therefore, we selected APA genes detected by at least six methods and also detected in the 3′ seq or bulk RNA-seq data. We then used the *get3UTRAPApd* function of movAPA (15) (with parameters *minDist* = 50, *minRatio* = 0.05) to determine the proximal and distal 3′ UTR pAs for each gene. Next, we calculated the differences in proximal and distal PAU (defined as △PAU) for each gene estimated by each method across different cell types, and compared these with the results based on bulk 3′ seq or bulk RNA-seq data. We selected the top 60% of genes with the smallest △PAU differences between the scRNA-seq and bulk data and calculated the Pearson correlation of △PAU for these genes.

Evaluation of DEAPA gene identification

Datasets for DEAPA evaluation

From the 17 datasets with cell-type annotations, we selected datasets for DEAPA analysis that met the following three criteria:

1) All methods yielded valid pA quantification results in the dataset;

2) The number of cells in each compared cell group was greater than 200;

3) The number of DEAPA genes identified by the majority of methods (≥4) exceeded 10.

In total, 8 datasets fulfilled these requirements and were used for the final DEAPA evaluation.

Construction of the consensus DEAPA gene set

Since true DEAPA genes are unknown in real data, we proposed a multi-level weighted strategy to construct a consensus DEAPA gene set as a reference for evaluation. This strategy selects high-confidence consensus DEAPA genes through gene ranking normalization and WF (Weighted Frequency) statistics, which can reduce the false positive rate and address the issue of significant differences in the number of DEAPA genes from different methods. Consensus DEAPA genes are defined as DEAPA genes identified by ≥ four methods and with a WF ≥ 1.6. The specific steps are as follows.

1) DEAPA gene identification

To assess the performance of methods in identifying DEAPA genes, we employed two differential analysis strategies: DEXseq and Wilcoxon rank-sum test. By randomly assigning each cell group into six pseudo-bulk subgroups, DEXSeq was used to identify APA sites showing differential usage between the two cell groups (adjusted *P*-value < 0.05 and |Fold Change| >1.5). The corresponding genes were designated as candidate DEAPA genes. For the Wilcoxon rank-sum test, we based on three APA usage indices—RUD, RUP, and PSI—to detect genes showing differential APA usage between two cell groups (adjusted *P*-value < 0.05 and |ΔRUD|, |ΔRUP|, or |ΔPSI| > 0.1). The APA usage of each gene in each single cell is quantified as RUD (Eq. 5), RUP (Relative Usage of the Proximal site), and PSI (ψ) (26,27), respectively. The following metrics are defined:

$$RUP_{s,i} = \frac{E_{p,i}}{\sum E_i} \tag{8}$$

The RUP for gene *i* in cell *s* is calculated as the ratio of the read counts for proximal pA to the total read counts for all 3' UTR pAs on that gene. The RUP score ranges between 0 and 1, with a higher RUP indicating increased use of the proximal site, *i.e.* shortening of the 3' UTR.

$$PSI_{s,i} = \frac{\sum_{k=1}^{n_i} E_{k,i} \cdot \left(\frac{k-1}{n_i-1}\right)}{\sum_{k=1}^{n_i} E_{k,i}} \tag{9}$$

The PSI for gene *i* in cell *s* is calculated as the weighted ratio of read counts for all 3' UTR pAs on that gene, where the weight is determined by the relative position of each site from upstream to downstream within the 3' UTR (0 for the most proximal site, 1 for the most distal site). The PSI score ranges between 0 and 1, with 0 representing exclusive usage of the proximal pA and 1 representing exclusive usage of the distal pA.

2) Standardization and ranking

For each gene, we ranked them in ascending order of adjusted *P*-value and descending order of |log2FC| based on the pAs with the most significant differences, and calculated the PR (Percentile Rank).

3) Determination of regulatory direction

We preliminarily determined the regulatory direction (3′ UTR shortened or lengthened) of DEAPA genes to avoid including genes in the core gene set that have high significance rankings across

multiple methods but completely opposite regulatory directions. The *n* pAs for each gene were sorted according to their distance from the stop codon, with the closest being ranked as 1 and the furthest as *n*. The pA ranked as 1 was considered the proximal pA, and the remaining pAs were considered distal pAs. For each DEAPA gene, we only focused on the differential usage of pA with the maximum |log2FC|. If it was a proximal pA and log2FC < 0, it was classified as lengthened; otherwise, as shortened. If it was a distal pA and log2FC < 0, it was classified as shortened; otherwise, as lengthened. This approach subdivided DEAPA genes into lengthened and shortened DEAPA genes.

4) Calculation of the WF score

For each DEAPA gene, we counted the number of methods that identified it and calculated the frequency score weighted by the PR. That is, for each gene, we accumulated the contribution values from all methods that identified it, where the contribution from each method is determined by (1 - PR). Finally, we selected genes with WF ≥ 1.6 and supported by ≥ four methods as the final consensus DEAPA genes.

$$WF_{gene} = \sum_{t=1}^{T}\left(1 - PR_{gene,t}\right) \times I\left(\mathrm{DEAPA}_{gene,t}\right) \tag{10}$$

$PR_{gene,t}$ represents the significance percentile rank of a gene in the differential analysis results obtained from the pA expression counts quantified by the tth method. I $(\mathrm{DEAPA}_{gene,t})$ is an indicator function that takes the value of 1 if the tth method identifies a gene as a DEAPA gene; otherwise, it takes the value of 0. The WF score ranges from [0, *T*] (where T is the total number of methods) and represents the composite confidence level for a gene consistently identified as a DEAPA gene by multiple methods. A higher score indicates a greater likelihood that the gene is a genuine DEAPA gene.

Metrics for evaluating DEAPA gene identification

We used the consensus DEAPA gene set as the ground truth and evaluated the accuracy of detected DEAPA genes by calculating precision and recall. To assess consistency in DEAPA gene identification between methods, we calculated the Jaccard index among DEAPA genes and the

Spearman correlation coefficient of their significance rankings. The precision, recall and Jaccard index for DEAPA gene identification are defined as follows.

$$Precision_{DE\ APA} = \frac{TP_{DE\ APA}}{TP_{DE\ APA} + FP_{DE\ APA}} \quad (11)$$

$$Recall_{DE\ APA} = \frac{TP_{DE\ APA}}{TP_{DE\ APA} + FN_{DE\ APA}} \quad (12)$$

$$Jaccard_{DE\ APA} = \frac{TP_{DE\ APA}}{TP_{DE\ APA} + FP_{DE\ APA} + FN_{DE\ APA}} \quad (13)$$

TP_{DEAPA} represents the number of genes identified as DEAPA genes that are also present in the consensus DEAPA gene set. FP_{DEAPA} represents the number of genes identified as DEAPA genes but absent from the consensus DEAPA gene set. FN_{DEAPA} represents the number of genes that are present in the consensus DEAPA gene set but have not been identified as DEAPA genes by the method.

References

1. Frankish, A., Diekhans, M., Ferreira, A.-M., Johnson, R., Jungreis, I., Loveland, J., Mudge, J.M., Sisu, C., Wright, J., Armstrong, J. *et al.* (2019) GENCODE reference annotation for the human and mouse genomes. *Nucleic Acids Res.*, **47**, D766-D773.
2. Shulman, E.D. and Elkon, R. (2019) Cell-type-specific analysis of alternative polyadenylation using single-cell transcriptomics data. *Nucleic Acids Res.*, **47**, 10027-10039.
3. Heinz, S., Benner, C., Spann, N., Bertolino, E., Lin, Y.C., Laslo, P., Cheng, J.X., Murre, C., Singh, H. and Glass, C.K. (2010) Simple combinations of lineage-determining transcription factors prime cis-regulatory elements required for macrophage and B cell identities. *Mol. Cell*, **38**, 576-589.
4. Scrucca, L., Fop, M., Murphy, T.B. and Raftery, A.E. (2016) mclust 5: Clustering, Classification and Density Estimation Using Gaussian Finite Mixture Models. *The R journal*, **8**, 289-317.
5. Kowalski, M.H., Wessels, H.-H., Linder, J., Dalgarno, C., Mascio, I., Choudhary, S., Hartman, A., Hao, Y., Kundaje, A. and Satija, R. (2024) Multiplexed single-cell characterization of alternative polyadenylation regulators. *Cell*, **187**, 4408-4425.e4423.
6. Patrick, R., Humphreys, D.T., Janbandhu, V., Oshlack, A., Ho, J.W.K., Harvey, R.P. and Lo, K.K. (2020) Sierra: discovery of differential transcript usage from polyA-captured single-cell RNA-seq data. *Genome Biol.*, **21**, 167.
7. Wu, X., Liu, T., Ye, C., Ye, W. and Ji, G. (2021) scAPAtrap: identification and quantification of alternative polyadenylation sites from single-cell RNA-seq data. *Brief. Bioinform.*, **22**.
8. Zhou, R., Xiao, X., He, P., Zhao, Y., Xu, M., Zheng, X., Yang, R., Chen, S., Zhou, L., Zhang, D. *et al.* (2022) SCAPE: a mixture model revealing single-cell polyadenylation diversity and

cellular dynamics during cell differentiation and reprogramming. *Nucleic Acids Res.*, **50**, e66.
9. Li, W.V., Zheng, D., Wang, R. and Tian, B. (2021) MAAPER: model-based analysis of alternative polyadenylation using 3' end-linked reads. *Genome Biol.*, **22**, 222.
10. Wang, R., Nambiar, R., Zheng, D. and Tian, B. (2018) PolyA_DB 3 catalogs cleavage and polyadenylation sites identified by deep sequencing in multiple genomes. *Nucleic Acids Res.*, **46**, D315-D319.
11. Li, G.-W., Nan, F., Yuan, G.-H., Liu, C.-X., Liu, X., Chen, L.-L., Tian, B. and Yang, L. (2021) SCAPTURE: a deep learning-embedded pipeline that captures polyadenylation information from 3′ tag-based RNA-seq of single cells. *Genome Biol.*, **22**.
12. Kang, B., Yang, Y., Hu, K., Ruan, X., Liu, Y.-L., Lee, P., Lee, J., Wang, J. and Zhang, X. (2023) Infernape uncovers cell type–specific and spatially resolved alternative polyadenylation in the brain. *Genome Res.*, **33**, 1774-1787.
13. Fansler, M.M., Mitschka, S. and Mayr, C. (2024) Quantifying 3′UTR length from scRNA-seq data reveals changes independent of gene expression. *Nat. Commun.*, **15**.
14. Fu, R., Riemondy, K.A., Sheridan, R.M., Hesselberth, J.R., Jordan, C.T. and Gillen, A.E. (2022) scraps: an end-to-end pipeline for measuring alternative polyadenylation at high resolution using single-cell RNA-seq. *bioRxiv preprint*.
15. Ye, W., Liu, T., Fu, H., Ye, C., Ji, G. and Wu, X. (2021) movAPA: modeling and visualization of dynamics of alternative polyadenylation across biological samples. *Bioinformatics*, **37**, 2470-2472.
16. Sherstnev, A., Duc, C., Cole, C., Zacharaki, V., Hornyik, C., Ozsolak, F., Milos, P.M., Barton, G.J. and Simpson, G.G. (2012) Direct sequencing of Arabidopsis thaliana RNA reveals patterns of cleavage and polyadenylation. *Nat. Struct. Mol. Biol.*, **19**, 845-852.
17. Zhang, R., Kuo, R., Coulter, M., Calixto, C.P.G., Entizne, J.C., Guo, W., Marquez, Y., Milne, L., Riegler, S., Matsui, A. *et al.* (2022) A high-resolution single-molecule sequencing-based Arabidopsis transcriptome using novel methods of Iso-seq analysis. *Genome Biol.*, **23**, 149.
18. Agarwal, V., Lopez, D., S., Kelley, D.R. and Shendure, J. (2021) The landscape of alternative polyadenylation in single cells of the developing mouse embryo. *Nat Commun*, **12**, 5101.
19. Li, S., Wang, Z., Hu, Y., Ni, Q., Feng, C., Hu, Y., Zhang, S. and Chen, M. (2024) Benchmarking alternative polyadenylation detection in single-cell and spatial transcriptomes. *bioRxiv preprint*.
20. Kang, L., Zhang, Q., Qian, F., Liang, J. and Wu, X. (2025) Benchmarking computational methods for detecting spatial domains and domain-specific spatially variable genes from spatial transcriptomics data. *Nucleic Acids Res.*, **53**, gkaf303.
21. Arefeen, A., Xiao, X. and Jiang, T. (2019) DeepPASTA: deep neural network based polyadenylation site analysis. *Bioinformatics*, **35**, 4577-4585.
22. Ye, W., Cheng, X., Bi, X. and Wu, X. (2026) PolyAseqTrap: a universal tool for genome-wide identification and quantification of polyadenylation sites from different 3′ end sequencing data. *Genome Biol.*, **27**, 65.

23. Shah, A., Mittleman, B.E., Gilad, Y. and Li, Y.I. (2021) Benchmarking sequencing methods and tools that facilitate the study of alternative polyadenylation. *Genome Biol.*, **22**.
24. Bryce-Smith, S., Burri, D., Gazzara, M.R., Herrmann, C.J., Danecka, W., Fitzsimmons, C.M., Wan, Y.K., Zhuang, F., Fansler, M.M., Fernández, J.M. *et al.* (2023) Extensible benchmarking of methods that identify and quantify polyadenylation sites from RNA-seq data. *RNA*, **29**, 1839-1855.
25. Robinson, M.D., McCarthy, D.J. and Smyth, G.K. (2010) edgeR: a Bioconductor package for differential expression analysis of digital gene expression data. *Bioinformatics*, **26**, 139-140.
26. Lee, S., Chen, Y.-C., Gillen, A.E., Taliaferro, J.M., Deplancke, B., Li, H., Lai, E.C. and Consortium, F.C.A. (2022) Diverse cell-specific patterns of alternative polyadenylation in Drosophila. *Nat. Commun.*, **13**, 5372.
27. Goering, R., Engel, K.L., Gillen, A.E., Fong, N., Bentley, D.L. and Taliaferro, J.M. (2021) LABRAT reveals association of alternative polyadenylation with transcript localization, RNA binding protein expression, transcription speed, and cancer survival. *BMC Genomics*, **22**, 476.
